# Genome-wide species delimitation analyses of a silverside fish species complex in central Mexico indicate taxonomic over-splitting

**DOI:** 10.1186/s12862-022-02063-0

**Published:** 2022-09-14

**Authors:** Victor Julio Piñeros, Carmen del R. Pedraza-Marrón, Isaí Betancourt-Resendes, Nancy Calderón-Cortés, Ricardo Betancur-R, Omar Domínguez-Domínguez

**Affiliations:** 1 Laboratorio de Ecología Molecular, Escuela Nacional de Estudios Superiores Unidad Morelia, Universidad Nacional Autónoma de México, Antigua Carretera a Pátzcuaro 8701, Ex-Hacienda de San José de La Huerta, 58190 Morelia, Michoacán Mexico; 2 Department of Biology, The University of Oklahoma, 730 Van Vleet Oval, Norman, OK 73019 USA; 3 Facultad de Ciencias Naturales, Universidad Autónoma de Querétaro, Avenida de Las Ciencias S/N Juriquilla, Delegación Santa Rosa Jáuregui, 76230 Querétaro, Mexico; 4 Laboratorio de Biología Acuática, Facultad de Biología, Universidad Michoacana de San Nicolás de Hidalgo, Edificio “R” Planta Baja, Ciudad Universitaria, 58030 Morelia, Michoacán Mexico; 5 Laboratorio Nacional de Análisis y Síntesis Ecológica Para la Conservación de Recursos Genéticos de México, Escuela Nacional de Estudios Superiores, Unidad Morelia, Universidad Nacional Autónoma de México, Apartado Postal 27-3 (Xangari), 58089 Michoacán Morelia, Mexico

**Keywords:** *Chirostomahumboldtianum*, Freshwater fishes, Genome-wide data, Genomic structure, SNP loci, Species delimitation

## Abstract

**Background:**

Delimiting species across a speciation continuum is a complex task, as the process of species origin is not generally instantaneous. The use of genome-wide data provides unprecedented resolution to address convoluted species delimitation cases, often unraveling cryptic diversity. However, because genome-wide approaches based on the multispecies coalescent model are known to confound population structure with species boundaries, often resulting in taxonomic over-splitting, it has become increasingly evident that species delimitation research must consider multiple lines of evidence. In this study, we used phylogenomic, population genomic, and coalescent-based species delimitation approaches, and examined those in light of morphological and ecological information, to investigate species numbers and boundaries comprising the *Chirostoma* “*humboltianum* group” (family Atherinidae). The *humboltianum* group is a taxonomically controversial species complex where previous morphological and mitochondrial studies produced conflicting species delimitation outcomes. We generated ddRADseq data for 77 individuals representing the nine nominal species in the group, spanning their distribution range in the central Mexican plateau.

**Results:**

Our results conflict with the morphospecies and ecological delimitation hypotheses, identifying four independently evolving lineages organized in three geographically cohesive clades: (i) *chapalae* and *sphyraena* groups in Lake Chapala, (ii) *estor* group in Lakes Pátzcuaro and Zirahuén, and (iii) *humboltianum* sensu stricto group in Lake Zacapu and Lerma river system.

**Conclusions:**

Overall, our study provides an atypical example where genome-wide analyses delineate fewer species than previously recognized on the basis of morphology. It also highlights the influence of the geological history of the Chapala-Lerma hydrological system in driving allopatric speciation in the *humboltianum* group.

**Supplementary Information:**

The online version contains supplementary material available at 10.1186/s12862-022-02063-0.

## Background

Species delimitation, which consists of determining the boundaries between species, is a fundamental aim of evolutionary biology, not only to document the extent of organismal diversity, but also to conserve and manage biodiversity. Species delimitation is challenging, as speciation is generally not instantaneous, where there is a diffuse area between populations and species—known as the speciation continuum [1]. The inference of species delimitation thus becomes particularly challenging in recently diverged or closely related species, where it is difficult to differentiate the population-level structure from distinct species as they often undergo incomplete lineage sorting (ILS) or hybridization [2].

The use of next generation sequencing techniques offers the opportunity to generate robust datasets consisting of many samples at thousands of loci to address species delimitation conflicts with unprecedented resolution, allowing for the detection of genetic structure at a finer scale [3, 4]. Coupled with species delimitation approaches under the multispecies coalescent (MSC) model, genome-wide data provide considerable statistical power to identify recently differentiated species boundaries [4]. However, the MSC model has been recently questioned as it may confound population structure with putative species, often leading to overestimating species diversity [4–6]. Thus, the implementation of MSC analyses combined with other approaches, such as phylogenomic and population genomic methods (e.g., multivariate, assignment, and genetic differentiation analyses), in conjunction with other lines of evidence (e.g., biogeographic, ecological, or life-history), confers a robust framework to assess species delimitations [7].

**Fig. 1:**
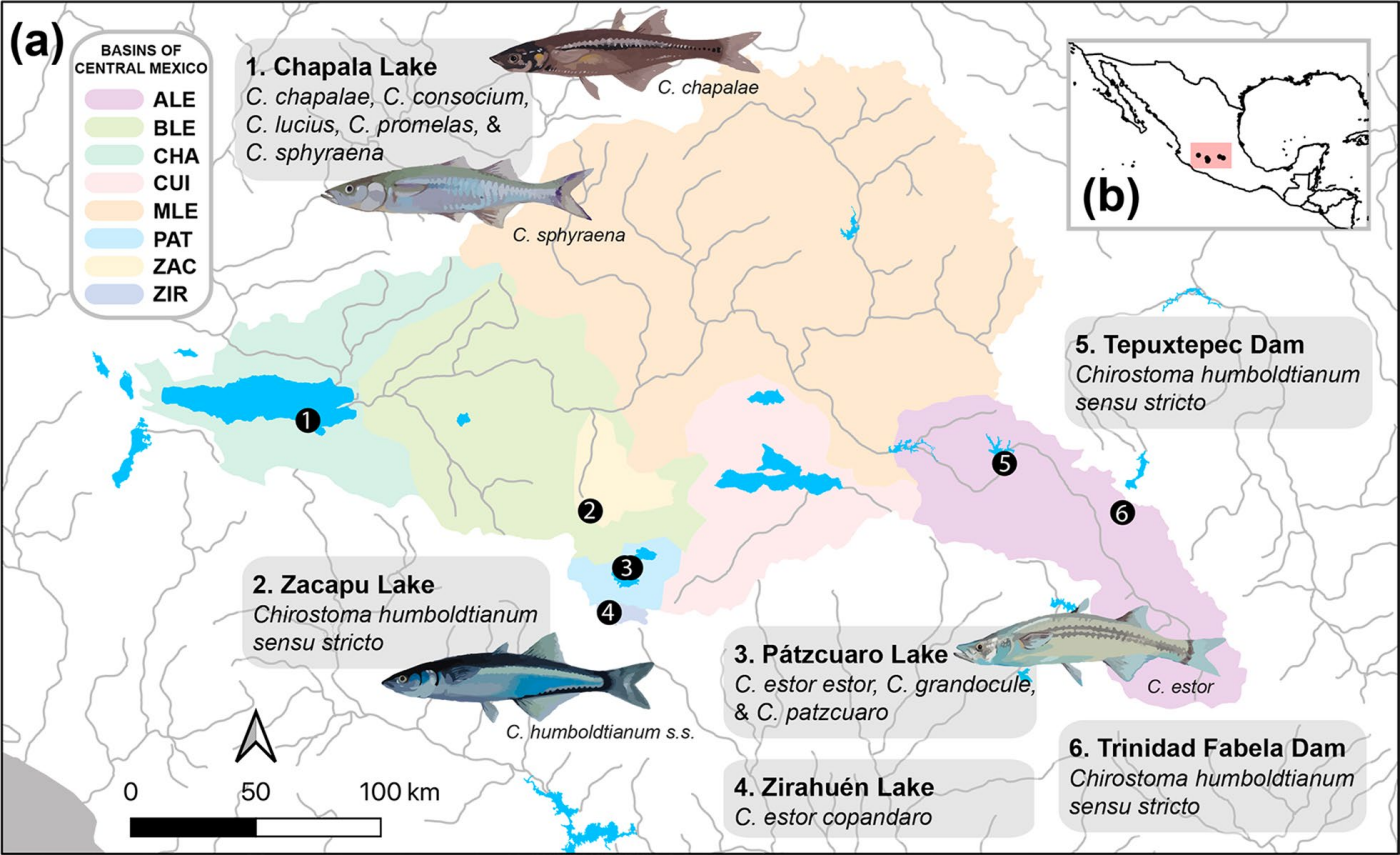
**a** Sampling localities of the *humboldtianum* group across the central Mexico plateau: Lake Chapala, Lake Zacapu, Lake Zirahuén, Lake Pátzcuaro, Tepuxtepec Dam, Trinidad Fabela Dam Basins CHA, Chapala; ZIR, Zirahuén; PAT, Pátzcuaro; ZAC, Zacapu; ALE, Alto Lerma; BLE, Bajo Lerma; MLE, Medio Lerma [126]. **b** Location of central Mexico plateau in Mexico. Artistic fish illustration credit: Sergio Godínez-Marrón

The New World silverside fishes in the *Chirostoma* *humboldtianum* species complex (Atherinopsidae; hereafter referred to as the “*humboldtianum* group”), distributed in central Mexico, have been of high economic and cultural importance since pre-Hispanic times [8]. Currently, *Chirostoma* species are considered one of the most important fishery resources in the region, where they have been severely overexploited [8, 9]. The *humboldtianum* group represents an interesting system in terms of species delimitation as their nominal species have been a subject of taxonomic controversy, with varying numbers of species recognized based on morphologic [10] or molecular [11] data. This group is geographically restricted to the lacustrine environments of the central Mexico plateau occurring mainly in Lakes Chapala, Pátzcuaro, Zirahuén, and Zacapu [10] (Fig. 1)––an area comprising roughly 1716.5 km^2^ [12, 13]––making it feasible to address species delimitation by examining the extent of species diversity based on samples collected across their range.

Nine nominal species in the *humboldtianum* group have been described based on morphological, osteological, meristic, and allozyme data: *C.* *chapalae*, *C.* *lucius*, *C.* *promelas*, *C.* *sphyraena*, and *C.* *consocium* from Lake Chapala; *C.* *grandocule*, and *C.* *patzcuaro* from Lake Pátzcuaro; *C.* *humboldtianum* sensu stricto from Lake Zacapu and the Lerma River System; and two subspecies for *C.* *estor*, *C.* *e.* *estor*, and *C.* *e.* *copandaro* from Lakes Pátzcuaro and Zirahuén, respectively [10, 14–17] (Fig. 1). While a molecular study using a single mitochondrial marker (NADH dehydrogenase subunit 2; *ND2*) failed to identify support for the monophyly for each of the nine nominal species, and also to resolve the phylogenetic relationships within the species complex [18], a recent phylogeographic study based on two mitochondrial (cytochrome b, *Cytb*; and a fragment of the hypervariable control region, *D-loop*) and one nuclear marker (first intron of the S7 ribosomal protein) identified five genetic groups, suggesting that the diversity in this species complex may be overestimated [11]. The genetic structure in these groupings largely clusters by lakes, indicating that diversification in this group is the result of historical geological and hydrographic processes.

Ecological divergence has also been suggested as a driver of speciation in this group given the high morphological disparity across traits related to different habitats [18, 19]. For instance, ecotypes (‘peces blancos’ and ‘charales’; 117–300 mm and 70–142 mm standard length SL, respectively) based on the size of the species could coexist by feeding on prey of different sizes [10]. Betancourt-Resendes et al. [11] observed intra-lacustrine patterns of evolution in Lakes Chapala and Pátzcuaro (two well-differentiated genetic groups within the Lake Chapala representing five morphospecies, and two genetic groups in Lake Pátzcuaro representing three morphospecies), suggesting that these lineages probably evolved through ecological speciation. Although no strong evidence of segregation of the nominal species by trophic specialization has been reported, these patterns remain to be evaluated [14, 20, 21]. The discrepancy in the recognition of nine morphological *versus* five mitonuclear species (sensu [11]), together with the economic importance and critical conservation status of these species, highlight the necessity of an accurate estimation of species boundaries in this group.

Here, we used double-digest restriction site-associated DNA sequencing (ddRADseq) to generate genome-wide single nucleotide polymorphism (SNP) data from all nine nominal species in the *humboldtianum* group, sampled throughout their distribution range. We also considered previous studies to interpret the observed genetic structure in the light of multiple lines of evidence. By generating this molecular dataset our study aims to: (i) test whether the morphospecies or ecotypes are concordant with the genomic groupings, (ii) investigate the number and boundaries of species in the *humboldtianum* group, (iii) examine the evolutionary processes driving the divergence in this species complex, and (iv) discuss conservation implications in light of the proposed species boundaries and their observed genetic structure. Ultimately, our study adds to the growing body of work addressing complex species delimitation scenarios with genomic data, while providing critical information to guide conservation and management efforts in the *humboldtianum* group.

## Results

### ddRADseq assembly and datasets

We obtained ~ 3.6 × 10^8^ sequence raw reads, of which ~ 3.5 × 10^8^ (97.6%) passed quality filters. On average, there were *ca*. 2 million reads per sample. The de novo assembly using a combination of *m*5*M*4*n*5 parameters generated 275,533 putative loci with an average coverage of 16.1 per sample. After filtering for missing data and different *maf* thresholds, we generated five final matrices (A–E), which varied from 1887 to 33,716 SNPs and 7.7–15.77% of missing data. These final matrices were also filtered to contain neutral-only and outlier loci, ranging from 1795 to 33,346 and 82–370 SNP loci, respectively. For more information, refer to the “Methods” and Additional file 1, 2 and 3.

### Multivariate analyses

**Fig. 2:**
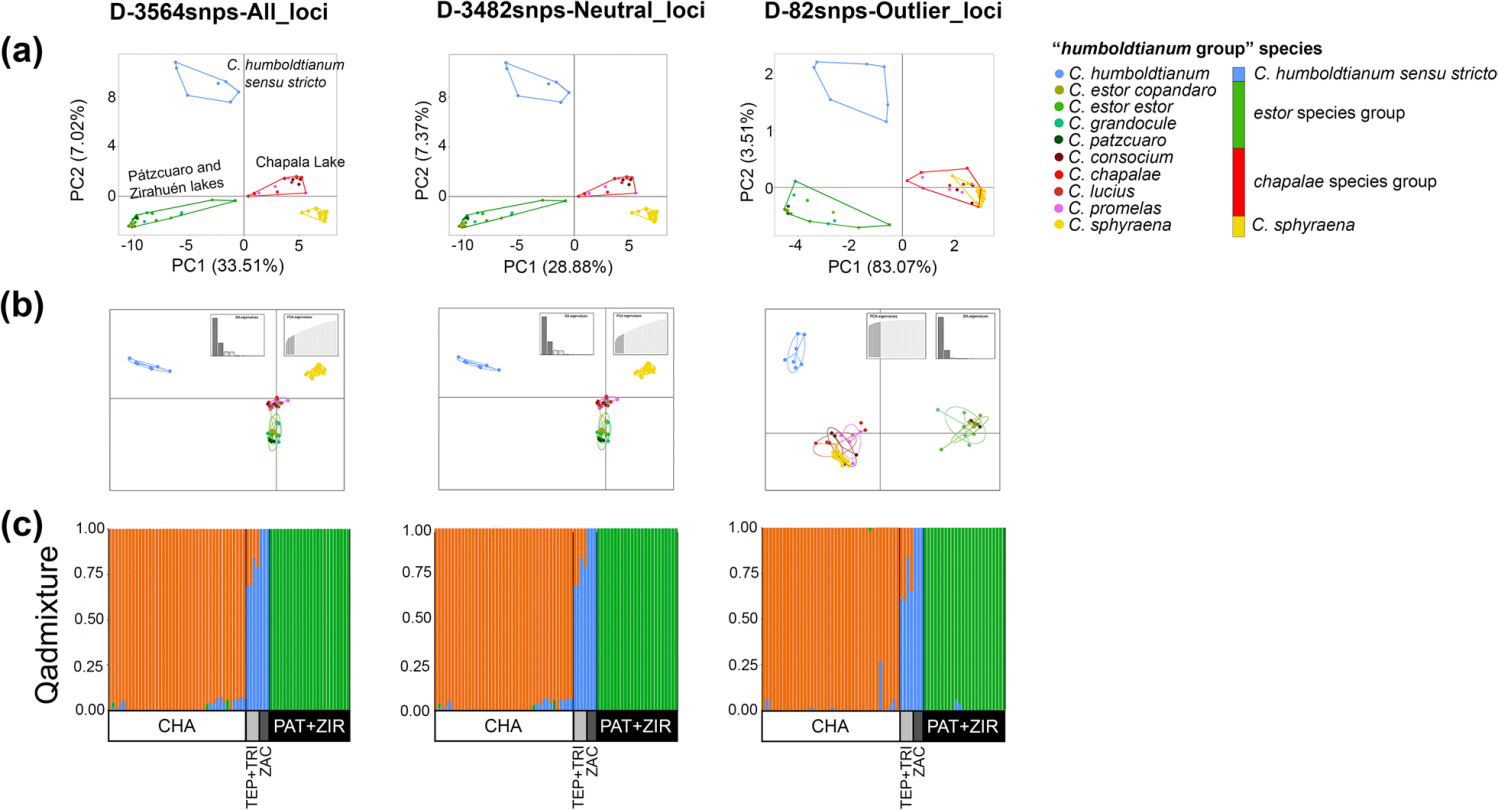
Multivariate and admixture results based on all, neutral, and outlier SNP loci from the matrix D (3564-snps loci), which explain the highest percentage of explained variation in the analyses. **a** Principal component analyses (PCAs), and (**b**) Discriminant analyses of principal components (DAPCs) consistently recovered four genomic groups (k = 4) with all and neutral loci that are in agreement with geographic patterns but not with the previously recognized morphospecies: *humboldtianum* sensu stricto group (blue), from Lake Zacapu; *estor* group (green) from Lakes Patzcuaro and Zirahuén; *chapalae* group (red), from Lake Chapala; and *C.* *sphyraena* group (yellow), also from Lake Chapala. Outlier loci resolved three groups (k = 3) where *chapalae* and *sphyraena* groups clustered together. Morphospecies are color-coded according to the genomic groups observed. In PCAs scatterplots the point clustering groups are delimited by convex hulls. In DAPC scatterplots, the point clustering groups are inside their 95% inertia ellipses, and the lines connect points to the mean value for each group. The eigenvalue bar plots are showing in the upper right of each figure. **c** Admixture assignment analyses estimated using all, neutral, and outlier SNPs consistently identified three well-differentiated clusters (k = 3). Each bar represents the probability of assignment to each cluster. Genomic clusters are color-coded as blue, *humboldtianum* sensu stricto, green, *estor* group; orange, *chapalae-sphyraena* group. CHA, Lake Chapala; TEP, Tepuxtepec Dam; TRI, Trinidad Fabela Dam; PAT, Lake Pátzcuaro; ZIR, Lake Zirahuén

The PCAs performed to evaluate the robustness of the genomic results consistently identified four genomic groups regardless of the number of SNPs (1887–33,716), individuals (37–72), morphospecies (4–9), or missing data (0.3–52.1%) included in each of the matrices (Additional file 2: Fig. S1). The PCAs including either all or neutral-only SNP loci (matrices A–E) also recovered four genomic groups, which align with the geography of the central Mexico plateau, but not with a delineation according to the previously recognized morphospecies. The first group (*humboldtianum* sensu stricto group hereafter) clustered samples from Lake Zacapu, Tepuxtepec, and Trinidad Fabela dams. The second group (*estor* group hereafter) included individuals of *C.* *e.* *estor,* *C.* *e.* *copandaro*, *C.* *grandocule*, and *C.* *patzcuaro* from Lakes Pátzcuaro and Zirahuén. The third group (*chapalae* group hereafter) is composed of samples of *C.* *chapalae*, *C.* *consocium*, *C.* *lucius,* and *C.* *promelas*, from Lake Chapala. Finally, the fourth group (*sphyraena* group hereafter) is formed by *C.* *sphyraena*, also from Lake Chapala. The first principal component accounted for 24.76–33.51% of the genetic variation and is congruent with the separation of *estor* and *humboldtianum* sensu stricto from *chapalae* and *sphyraena* groups. The second principal component represented 6.37–7.47% of the genetic variation, resulting in the clear segregation of the *humboldtianum* sensu stricto group (Fig. 2a; Additional file 2: Fig. S2).

While the results of the PCAs estimated using outlier SNP loci did not produce a clear demarcation relative to matrices considering all and neutral-only SNPs, these analyses mostly resulted in the same clustering patterns except in two instances (matrices D and E) where the *chapalae* and *sphyraena* groups clustered together. The first two principal components of the PCAs calculated with outlier SNPs accounted for 20.32–86.58% of the genomic variation explained. Overall, PCAs generated with just outlier (82–370) SNP loci delineated between three and four genomic clusters, superimposing populations from *chapalae* with *sphyraena* groups (Fig. 2a; Additional file 2: Fig. S2).

Scatter plots of the DAPC analyses produced results similar to those based on PCA. The first two discriminant axes identified the same four genomic clusters (BIC: *k* = 4) (Fig. 2b; Additional file 2: Figs. S3, S4), retaining 9–12 PCs that represent 48.5–57.7% of the cumulative variance. The DAPC analyses estimated using the outlier loci (matrices C, D and E) also identified three genomic clusters (BIC: *k* = 3) where *chapalae* and *sphyraena* form a single group (Fig. 2b; Additional file 2: Figs. S3, S4).

### Genomic structure and genomic differentiation analyses

Admixture assignment results of the SNP loci (matrices A–E) considering all, neutral-only, and outlier loci identified three genomic clusters (*k* = 3) as the best-fit model (Additional file 2: Fig. S5), each corresponding to the major groups (*humboldtianum* sensu stricto, *estor*, and *chapalae*-*sphyraena*) obtained with PCA and DAPC analyses using the outlier SNP loci (Fig. 2c; Additional file 2: Fig. S6). As in previous analyses, the admixture results did not delineate morphospecies boundaries.

All individuals of the *humboldtianum* sensu stricto group collected from artificial dams (three from Tepuxtepec and one from Trinidad Fabela) had strong admixture with the *chapalae* group (0.11–0.45), whereas *chapalae* and *estor* groups produced rather low values of shared genetic information (< 0.10). While there were higher levels of individual admixture between *humboldtianum* sensu stricto and *chapalae* groups when fewer SNP loci were analyzed, a higher number of outlier SNP loci (99–370) identified more admixture between the three groups than those considering fewer outlier SNPs (82–92) (Fig. 2c; Additional file 2: Fig. S6).

Intra-lake admixture analyses for Lake Chapala identified no evidence of finer genetic structure, as the best-fit model supports a scenario represented by a single metapopulation (*k* = 1, Additional file 2: Fig. S7), clustering all individuals of the *sphyraena* and *chapalae* groups. Genetic structure was not observed within the Pátzcuaro-Zirahuén lakes either (*k* = 1). By and large, the observed genetic structure based on intra-lake admixture analyses conflict with both morphospecies and ecotype groupings (‘peces blancos’ *vs.* ‘charales’) (Additional file 2: Fig. S7).

Pairwise *F*_ST_ comparisons were much higher and statistically more significant at inter-lacustrine than at intra-lacustrine levels, reflecting a strong association between genetic structure and geography (Additional file 3: Tables S4–S7). Pairwise *F*_ST_ values between the nine morphospecies varied from 0.023 to 0.256 and were mostly significant (*p* values < 0.0012, α = 0.0014) at inter-lacustrine comparisons, with the exception of *C.* *chapalae* which showed no significant differentiation with the rest of the morphospecies. By contrast, comparisons among sympatric morphospecies from Lakes Chapala and Pátzcuaro resulted in negative, non-significant values (*F*_ST_ = − 0.317 to – 0.012, *p* values > 0.029), except for *C.* *sphyraena* which was significantly different than the rest (Additional file 3: Table S4). A greater genetic differentiation was found between the five mitonuclear groups (0.050–0.246), where the majority of the pairwise comparisons were statistically significan (*p* values < 0.0001, α = 0.005), except for comparisons between subspecies *C.* *e.* *estor* and *C.* *e.* *copandaro* (*p* value = 0.05; Additional file 3: Table S5). *F*_ST_ values between DAPC clusters were significant and varied from 0.04–0.248 (*p* values < 0.0001, α = 0.0083; Additional file 3: Table S6); the three genomic groups detected by the admixture analyses also resulted in significant *F*_ST_ values (0.120–0.217, *p* values < 0.0001, α = 0.017; Additional file 3: Table S7). Finally, the genetic structure between ‘peces blancos’ and ‘charales’ ecotypes (− 0.005 to 0.001) was not significant in any of the intra-lacustrine comparisons (*p* values = 0.01–0.027, α = 0.005; Additional file 3: Table S8).

### Phylogenetic analyses

**Fig. 3:**
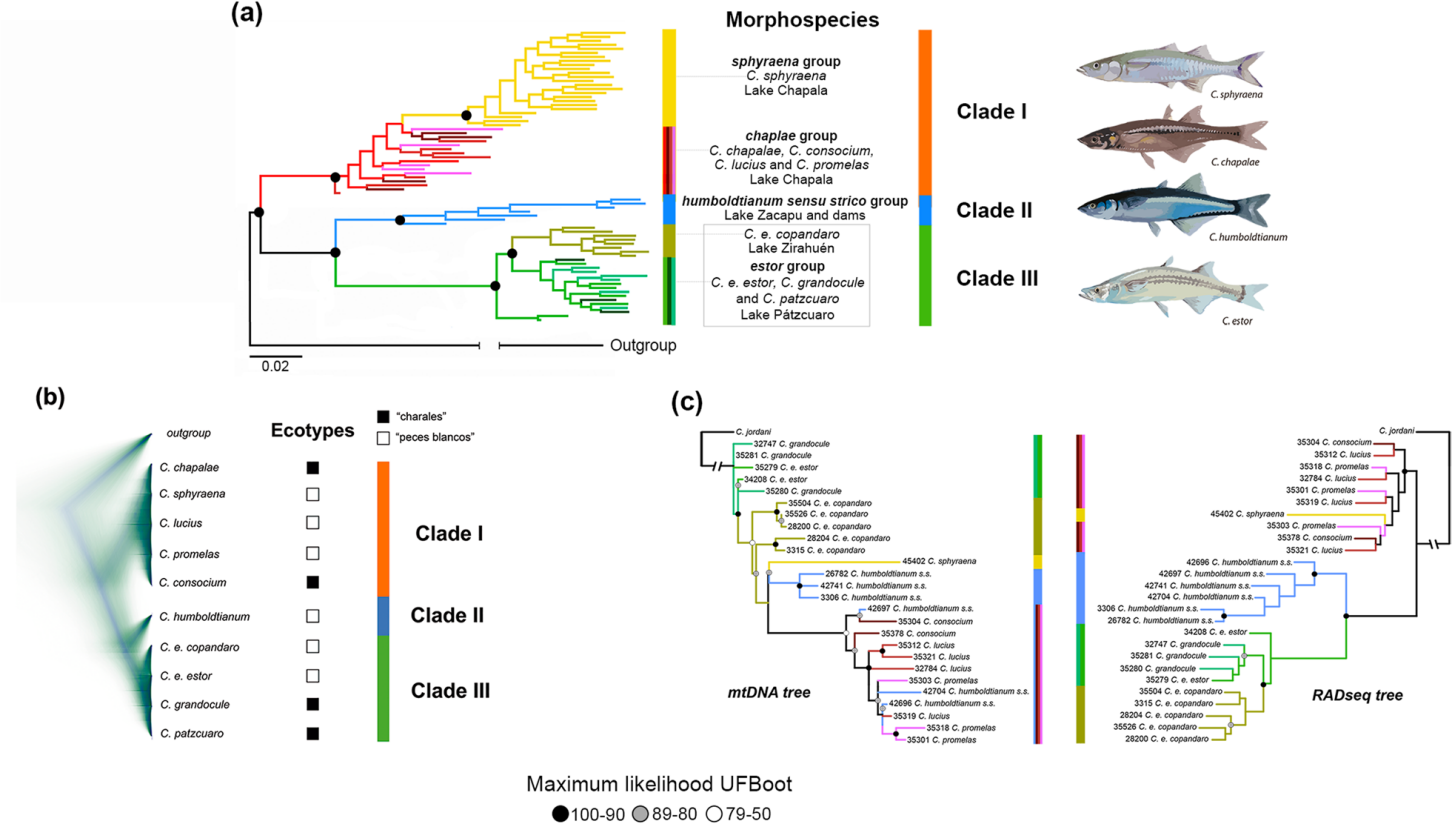
The ML phylogenetic tree based on 3564 SNP loci (**a**) recovered three well-differentiated clades: clade I formed by species in Lake Chapala, clade II represented by species from Lake Zacapu and dams, and clade III composed by species in Lakes Pátzcuaro and Zirahuén. We observed *C.* *sphyraena* as a monophyletic group within clade I, and the subspecies *C.* *e.* *copandaro* from Lake Zirahuén as a differentiated cluster within clade III. Although the multi-coalescent species tree considering circa 3400 neutral SNPs (**b**) recovered the main genomic clusters as the rest of the genetic structure analyses and the ML phylogenetic inferences, it did not present any intra-lake divergences. **c** The phylogenetic inference based on *mt*DNA (*Cytb* and *D-loop*) failed to delineate reciprocal monophyletic groups as the ones estimated using the ddRADseq data (3564 SNP loci). The analyses of the genome-wide datasets clearly recovered well-differentiated clusters that are in agreement with the geography of the central Mexico plateau. However, none of our phylogenetic inferences showed concordance with the morphospecies nor ecotypes recognized within the *humboltianum* group. Numbers on branches of the main clades indicate bootstrap values. Artistic fish illustration credit: Sergio Godínez-Marrón

Phylogenetic inferences in a Maximum Likelihood (ML) framework using IQ-TREE based on matrices A, C, D and E (all and neutral) SNP loci resolved three highly supported clades (ultrafast bootstrap or UFBoot = 90–99%; Figs. 3a and Additional file 3: Fig. S8), which are largely in agreement with the results from admixture analyses. Clade I clustered species within *chapalae* and *sphyraena* groups from Lake Chapala (UFBoot = 83–95%); clade II is formed by the *humboldtianum* sensu stricto group from Lake Zacapu and dams (UFBoot = 73–100%); and clade III is represented by the *estor* group from Lakes Pátzcuaro and Zirahuén (UFBoot = 100%). Samples of *C.* *sphyraena* and the subspecies *C.* *e.* *copandaro* were resolved as monophyletic within clade I (UFBoot = 80–100%) and clade III (UFBoot = 94–97%), respectively. While the phylogenetic trees obtained with outlier loci datasets had less resolution than those using the complete matrix, these different analyses resolved the reciprocal monophyly of Clade I and Clades II-III, except for matrix A where Clade I was paraphyletic. Also, the relationship between Clade II and III was more problematic: in some cases these clades were reciprocally monophyletic (matrices A and C), but in other cases they were paraphyletic (databases B, D, and E) (Additional file 2: Fig. S8).

Tree inference based on mitochondrial data failed to resolve the groups identified with our genome-wide RADseq analyses, suggesting mitonuclear discordance (Fig. 3c; Additional file 1: Fig. S9). However, in agreement with the ddRADseq phylogenies, analyses of the mitochondrial genealogies and a phylogeny estimated using the concatenated matrix did not delineate any clear monophyletic groups according to morphospecies boundaries. Although some groupings comprised of neighboring localities were resolved with the *mt*DNA trees, resulting clades from these trees are not cohesively clustered by geography. The *Cytb* genealogy resolved the monophyly of *C.* *sphyraena*, a result that is concordant with the RADseq phylogeny and previous studies [11]. However, these relationships were not resolved with confidence (UFBoot < 95%). Overall, none of the phylogenetic hypotheses were aligned with the morphospecies or ecotypes proposed within the *humboldtianum* group (Fig. 3; Additional file 2: Fig. S9).

### Multispecies coalescent analyses

The species tree estimated with SNAPP also evidenced three geographically cohesive clades (I, II, and III; Fig. 3b). Coalescent-based species delimitation analyses using the BFD* method selected species delimitation scenarios of four and five species as the top-ranked models with the highest MLE values (MLE = − 30,568.17 and − 21,025.86 to − 11,910.25, respectively; Table 1). The main difference of the models of four and five species relies on the subspecies *C.* *e.* *copandaro* from Lake Zirahuén forming a different group from *C.* *e.* *estor* and the sympatric species in Lake Pátzcuaro. Bayes factors analyses supported models of four (subset 1 and 3) and five species (subset 2), rejecting the nine species that reflects the current taxonomy (BFs = 295.56–850.28; Table 1) and also alternative models of three species (BFs = 158.5–660.4; Table 1).

**Table 1:** Results of bayes factor delimitation (BFD*) analyses for the *humboldtianum* group using three SNPs subsets (ranging from 39 to 59 individuals, and 411–1102 SNP loci)

Model	Species number	Subset 1: 39ind_411 SNP loci		Subset 2: 39ind_1102 SNP loci		Subset 3: 59ind_548 SNP loci	
		MLE	BF	MLE	BF	MLE	BF
Current morpho-species	9	− 12,058.03	295.56	− 30,993.31	850.28	− 21,279.57	507.42
Mitonuclear groups	5	**− 11,910.25**	NA	− 30,600.16	63.98	**− 21,025.86**	NA
DAPC groups	4	− 11,927.68	34.85	**− 30,568.17**	NA	− 21,161.43	271.14
Admixture groups	3	− 11,989.52	158.53	− 30,898.37	660.41	− 21,278.3	504.88

Bayes factor (BF) calculations are estimated against the model with the best marginal-likelihood estimate (Model 1). Positive BF values indicate support for model 1 (the best model). Overall, these analyses support a species delimitation scenario between four and five species, rejecting the nine morphospecies scheme

MLE: marginal likelihood estimates. Bold values correspond to the best model selected by the analysis. NA correspond to not applied, and its associated marginal likelihood is in bold. 0 < BF < 2, non-significant; 2 < BF < 6, positive evidence; 6 < BF < 10, strong support; BF > 10, decisive support

### Genetic diversity and effective population size (*Ne*) analyses

The genomic diversity of the *humboldtianum* group is summarized in Table 2. The observed and expected heterozygosity (*Ho* and *He*) across different lineages was 0.097–0.136 and 0.084–0.139, respectively. All lineages distributed within Lake Chapala revealed higher genetic diversity (*Ho* = 0.123–0.138) than those in the Lakes Zacapu and Pátzcuaro-Zirahuén (*Ho* = 0.100–0.126) (Table 2). The values of *Ne* estimator (*HetExcessNe* and *CoancentryNe*) across different lineages were 7.2–317 and 2.2–31, respectively (Table 2). Values of *LDNe* estimator were infinite with the exception of *chapalae-sphyraena* group (86.9), while *C.* *humboldtianum* sensu stricto produced infinite values with both *LDNe* and *HetExcessNe* estimators (Table 2). These infinite values indicate the effect of sampling error.

**Table 2:** Genomic diversity of 3482 neutral SNPs loci estimates for the *humboldtianum* group, under each species delimitation hypothesis examined in this study (i–iv)

	N	*HO* (SD)	*HE* (SD)	*F* (SD)	*LDNe* (95% CIs)	*HetExcessNe* (95% CIs)	*CoancestryNe* (95% CIs)
9 morphospecies (i; Barbour, 1973)							
*C.* *chapalae*	1	–	–	–	–	–	–
*C.* *consocium*	4	0.138 (0.004)	0.110 (0.003)	− 0.225 (0.008)	Infinite (infinite–infinite)	8.9 (6.4–14.9)	10.9 (8.1)
*C.* *lucius*	7	0.134 (0.004)	0.115 (0.003)	− 0.144 (0.007)	Infinite (infinite–infinite)	12.6 (8.4–25.6)	6 (4.9)
*C.* *promelas*	5	0.123 (0.004)	0.099 (0.003)	− 0.222 (0.009)	Infinite (infinite–infinite)	Infinite (20.1–infinite)	31 (19.3)
*C.* *sphyraena*	24	0.136 (0.004)	0.121 (0.003)	− 0.083 (0.005)	Infinite (infinite-infinite)	8.9 (7.2–11.6)	2.2 (1.9)
*C.* *humboldtianum*	7	0.126 (0.004)	0.117 (0.003)	− 0.062 (0.007)	Infinite (infinite–infinite)	Infinite (108.1–infinite)	3 (2.7)
*C.* *estor*	15	0.103 (0.004)	0.096 (0.003)	− 0.041 (0.007)	Infinite (infinite–infinite)	244 (19.9–infinite)	3.6 (3.2)
*C.* *grandocule*	6	0.100 (0.004)	0.084 (0.003)	− 0.170 (0.008)	Infinite (infinite–infinite)	10.6 (6.9–24.6)	7.2 (5.4)
*C.* *patzcuaro*	3	0.112 (0.004)	0.085 (0.003)	− 0.301 (0.009)	Infinite (infinite–infinite)	7.2 (5.3–11.8)	Infinite (infinite)
5 mitonuclear groups (ii, Betancourt-Resendes et al*.* 2020)							
*C.* *chapalae*	17	0.130 (0.003)	0.139 (0.003)	0.079 (0.007)	Infinite (infinite–infinite)	24.7 (13.5–179.5)	4.5 (3.9)
*C.* *sphyraena*	24	0.136 (0.004)	0.121 (0.003)	− 0.083 (0.005)	Infinite (infinite–infinite)	8.9 (7.2–11.6)	2.2 (1.9)
*C.* *humboldtianum*	7	0.126 (0.004)	0.117 (0.003)	− 0.062 (0.007)	Infinite (infinite–infinite)	Infinite (108.1–infinite)	3 (2.7)
*C.* *e.* *estor*	16	0.103 (0.004)	0.096 (0.003)	− 0.037 (0.007)	Infinite (infinite–infinite)	10.8 (7.6–18.9)	4.4 (3.6)
*C.* *e.* *copandaro*	8	0.097 (0.004)	0.105 (0.003)	0.082 (0.010)	Infinite (infinite–infinite)	8.9 (6.2–16.4)	5.4 (4.2)
4 multivariate DAPC clusters (iii, *This* *study*)							
*C.* *chapalae* group^a^	17	0.130 (0.003)	0.139 (0.002)	0.079 (0.007)	Infinite (infinite–infinite)	24.7 (13.5–179.6)	4.5 (3.9)
*C.* *sphyraena*	24	0.136 (0.004)	0.121 (0.003)	− 0.083 (0.005)	Infinite (infinite–infinite)	8.9 (7.2–11.6)	2.2 (1.9)
*C.* *humboldtianum* sensu stricto	7	0.126 (0.003)	0.117 (0.003)	− 0.062 (0.007)	Infinite (infinite–infinite)	Infinite (108.1–infinite)	3 (2.7)
*C.* *estor* group^b^	24	0.101 (0.003)	0.104 (0.003)	0.103 (0.008)	Infinite (infinite–infinite)	106.4 (20.2–infinite)	4.4 (3.8)
Admixture clusters K = 3 (iv, *This* *study*)							
C. *chapalae-sphyraena* groups	41	0.134 (0.003)	0.138 (0.003)	0.085 (0.006)	86.9 (84.8–89.1)	317 (40–infinite)	4.2 (3.8)
*C.* *humboldtianum* sensu stricto group	7	0.126 (0.003)	0.117 (0.003)	− 0.062 (0.007)	Infinite (infinite–infinite)	Infinite (108.1–infinite)	3 (2.7)
C. *estor* group	24	0.101 (0.003)	0.104 (0.003)	0.103 (0.008)	Infinite (infinite–infinite)	106.4 (20.2–infinite)	4.4 (3.8)

*N* number of individuals analyzed per group, observed (*H*_*O*_) and expected (*H*_*E*_) heterozygosity, fixation index coefficient (*F*), linkage disequilibrium effective (*LDNe*), heterozygote-excess (*HetExcessNe*), and molecular coancestry (*CoancestryNe*) population size estimators. SD = standard deviation; CI = confidence intervals. The grey shading in the cells indicates the highest values of genetic diversity

^a^*C*. c*hapalae* group conformed by *C.* *chapalae,* *C.* *consocium,* *C.* *lucius,* and *C.* *promelas*

^b^*C.* *estor* group represented by *C.* *e.* estor, *C.* *e.* *copandaro,* *C.* *grandocule*, and *C.* *patzcuaro*

### *FST* loci outlier analysis

A total of 410 outlier loci (1.1% of the total analyzed, Fig. S10) were compared to GenBank entries using BLAST-n. In summary, 116 sequences did not have a match, while the remaining 294 (71.7%) loci matched fish sequences (E-value = 4E−42 to 2E−02), including protein-coding genes (26%) (Additional file 3: Table S9). Out of these coding regions, we identified genes related to a wide array of biological functions such as genes implicated in immune responses (*kpna3*: *Nothobranchius* *furzeri*, GenBank accession number XM_015966110.1; *bcl11b*: *Cheilinus* *undulatus*, XM_041804421.1; *trim59*: *Megalops* *cyprinoides*, XM_036537431.1; *NLR* *family* *CARD* *domain-containing* *protein* *3-like*: *Acanthophagus* *latus*, XM_037091148.1), sensory systems (*or132-1*: *Danio* *rerio*, DQ306116.1*;* *sws2a* and *rh2-1*: *Lucania* *goodei*, MT850055.1; and *lws2*: *Monopterus* *albus*, XM_020592984.1), growth (*cand1*: *Poecilia* *formosa*, XM_007569621.2; and *sgta*: *Archocentrus* *centrarchus*, XM_030727116.1), and skeletal-muscle system (*neb*: *Gymnodraco* *acuticeps*, XM_034221710.1; and *ttn*: *Fundulus* *heteroclitusi*, XM_036135404.1) (Additional file 3: Table S9).

## Discussion

In this study, we used ~1800 to ~33,000 SNPs to test the morphological- (nine nominal species) and *mt*DNA/nDNA-based (five mitonuclear groups) hypotheses to elucidate the number and boundaries of species in the *humboldtianum* group. Our results consistently identified four independently evolving lineages organized in three well-differentiated clades: *chapalae*-*sphyraena* in Lake Chapala, *humboldtianum* sensu stricto in Lake Zacapu and dams, and *estor* in Lakes Pátzcuaro and Zirahuén. The genomic clusters obtained were not in agreement with the morphospecies delimitation but rather with geography reflecting ancient isolation events in central Mexico. This scenario suggests that the geologic history of the Lerma-Chapala hydrologic system has played a major role in driving divergence in this species complex. Our analyses also revealed an intra-lake cladogenetic event where *C.* *sphyraena* (‘pez blanco’) is distinguished from its sympatric counterparts (‘peces blancos’ and ‘charales’) in Lake Chapala. Although body size in *Chirostoma* species in Lake Chapala has been suggested as a promoter of ecological niche partitioning for this species complex [10, 21], we did not find evidence of genetic structure related to the ‘peces blancos’ and ‘charales’ ecotypes.

Herein, the use of genome-wide data provided an unprecedented resolution that had not been achieved using a scant number of genetic markers to test species delimitation scenarios within the *humboldtianum* group [11, 18]. While species delimitation studies examining thousands of genetic loci often unveil cryptic diversity [22–25], our study represents one of the few cases where the use of genome-wide SNP data and MSC approaches provide evidence of taxonomic over-splitting [2, 5, 26]. Recently, it has been recognized that the MSC model can potentially confound population structure with species boundaries—particularly when major sampling gaps near the distribution range exist—leading to an over-estimation of the number of species, a bias compounded by the high statistical power that often results from genome-wide analyses [4–6]. However, we account for these caveats by assessing our results in a framework that examines all nominal species in the *humboldtianum* group from across their distribution ranges in the Lerma-Chapala hydrologic system, and by taking into account morphological and ecological lines of evidence. All in all, we provide a robust analysis to assess the number of species within the *humboldtianum* group and also to examine the evolutionary history of the group.

### Inter-lacustrine divergences

Our results suggest that vicariance events during the Pleistocene influenced the early divergence within the *humboltianum* group. Speciation within this group appear to be strongly related to the complex geologic history of volcanism, tectonism, and climatic events that promoted the connection and disconnection of the Lerma-Chapala hydrological system, and surrounding tributaries—including several lakes and paleolakes such as Chapala, Cuitzeo, Zacapu, Pátzcuaro, and Zirahuén [10].

Although our phylogenetic results are somewhat incongruent with those previously estimated by Betancourt-Resendes et al. [11] (*chapalae*, *sphyraena*, *estor*, and *humboldtianum* sensu stricto groups), their divergence time analyses provide a rough estimate of the timing of cladogenesis in the group, placing the origin of independent evolutionary lineages within the *humboldtianum* group during the Pleistocene < 1 Ma (0.58–0.13 Ma). There are two main biogeographic processes that are synchronous and congruent with the observed genetic patterns recovered in this study, suggesting that these events played an important role as the main drivers of diversification in the *humboldtianum* group. First, the allopatric fragmentation of clade I (Lake Chapala) from II–III (Lake Zacapu-Lakes Pátzcuaro and Zirahuén) may correspond to a biogeographical barrier promoted by the geologic activity of the Penjamillo Graben and the formation of the Tarascan corridor, which started much earlier during the Late Miocene–Early Pliocene [27, 28]. These geological events separated the ancient corridors between the paleo Lerma-Chapala system and the Cuitzeo paleolake plus adjacent tributaries that connected Lakes Zacapu, Pátzcuaro, and Zirahuén [10, 29]. The separation of these hydrologic regions experienced its highest peak during the Early Pleistocene [29], where severe climatic fluctuations promoted a series of connection and disconnection events of small water bodies, with the last dry episode starting *ca.* 0.12 Ma [10, 29]. The second biogeographic event is the fragmentation of the ancestral Villa Morelos and Chucandiro-Huaniqueo corridors, also during the Pleistocene. This episode, promoted by the geologic activity of the Northeast-Southwest fault system *ca.* 0.7–0.5 Ma, separating Lake Zacapu from Lakes Cuitzeo-Pátzcuaro-Zirahuén [30, 31], and correlating with the genetic patterns of divergence between clade II (Lake Zacapu) and III (Lakes Pátzcuaro and Zirahuén).

The same cladogenetic patterns, in agreement with the aforementioned biogeographic events, have been observed in goodeid freshwater species endemic to central Mexico, including the divergence between the sister species *Skiffia* *multipunctata* and *S.* *lermae* and the split of the *Allotoca* *diazi* complex from *A.* *zacapuensis*, although these diversification events do not appear to have occurred in synchrony [32–35].

### Intra-lacustrine divergences

Our genome-wide data revealed the presence of two genetic clusters in Lake Chapala (*sphyraena* and *chapalae* groups), as suggested by Betancourt-Resendes et al. [11] using a few *mt*DNA and *n*DNA sequences. The evolutionary processes segregating these populations seem to be related to ecological speciation—divergent specializations promoted by ecological opportunity following reproductive isolation [36, 37]—a major driver of sympatric evolution in lakes [36, 38, 39]. The most iconic examples of ecological speciation are depicted by South American [40–42] and African cichlids [43, 44], and sticklebacks [45, 46], where patterns of morphological divergence are associated with trophic partitioning. For example, preference for soft mobile (e.g., copepods) compared to hard sessile preys (e.g., gastropods) can lead to disruptive selection on skull morphology and body shape [47], where body size can be crucial to succeed in the related foraging mode (e.g., benthic *vs.* limnetic in *Gasterosteus* spp; [48, 49]).

In the case of species of the *humboldtianum* group occurring within the Lake Chapala (*C.* *sphyraena*, *C.* *promelas*, *C.* *consocium*, and *C.* *lucius*), several hypotheses of ecological speciation suggest the coexistence of ecotypes in agreement with a differentiation of morphological traits (e.g., jaw shape, head length, oral gape, and gill raker structure) related to feeding habits [10, 50], or an ecological partition that correlates with species body sizes (larger ‘peces blancos’ vs*.* smaller ‘charales’) [10, 16, 21]. However, no strong evidence showing clear patterns of differentiation between morphospecies or ecotypes has been documented to date [21].

Herein, we evaluated the hypothesis by Barbour [10] that differentiation in body length would promote niche partitioning, allowing the co-occurrence of different species that feed on distinct prey sizes. Although signals of trophic specialization separating ‘peces blancos’ and ‘charales’ have been documented by Mercado-Silva et al. [21] for three species in Lake Chapala, no analysis conducted here (PCAs, DAPCs, inter- and intra-lake admixture, and phylogenetic trees; Figs. 2 and 3; Additional file 2: Figs. S1–S9) provide support for a scenario of diversification related to body size. Nonetheless, our multivariate and *F*_ST_ analyses (Fig. 2a, b, Additional file 2: Figs. S1, S2, S4; Additional file 3: Tables S4–S6) clearly demonstrate that the *sphyraena* group—the only entity previously resolved as monophyletic based on a scant number of mitochondrial and nuclear markers [11]—represents a separate genetic cluster with almost no gene flow shared with other members in the *chapalae* group, although in our intra and inter-lake admixture result did not find evidence of *sphyraena* and *chapalae* groups genetic separation (see “Discussion” below). Based on this evidence, and considering the fact that *C.* *sphyraena* is the most taxonomically differentiated nominal species (particularly on traits related to trophic specialization; [10]), we hypothesize that *C.* *sphyraena* is in its early stages of ecological speciation. We note, however, that species-specific habitats are unknown for most of the *Chirostoma* species [21], and thus further ecological studies are necessary to better understand the evolutionary history of the *chapalae* and *sphyraena* groups.

### Species delimitation and taxonomic implications

The BFD* species delimitation analyses provided strong support for four- and five-species delimitation scenarios (Table 1), while the coalescent-based species tree identified three major monophyletic groups (Fig. 3b). Recent simulation studies that evaluated the efficiency of MSC methods suggest that this model tends to confound population structure with species boundaries [4–6]. This becomes particularly problematic in recently-diverged and closely-related species, such as the *humboldtianum* group, where processes promoting differentiation lie at the intersection between population structure and species divergence [4], generating gene trees with short branches and with multi coalescent histories that make species tree inference challenging [51, 52]. This is not the case in the *humboldtianum* group, a recent species complex that diverged less than 1 Ma [11], where the genetic structure detected fewer species than those recognized by taxonomic studies.

The finer genetic structure recovered for the *sphyraena* group and the sub-species *C.* *e.* *copandaro* favor models of four or five species over a three-species scheme. In this scenario, under a strict reciprocal monophyly criterion, none of the *C.* *sphyraena* or the sub-species *C.* *e.* *copandaro* lineages would represent a species (under the phylogenetic species concept, PSC; [1]). However, our intra-lake admixture analyses suggest that there is no genetic structure among *sphyraena* and *chapalae* groups. We conducted intra-lake admixture analyses to evaluate whether including the rest of the species in the *humboldtianum* group may be potentially masking finer genetic structure within each lake. Our results demonstrate that the best-fit model for each lake is represented by a single population (Additional file 2: Fig. S7). These results, combined with the admixture analyses based on all individuals (matrices A–E; Figs. 2c; Additional file 2: Fig. S6) and phylogenetic trees (Fig. 3; Additional file 2: Fig. S8) suggest that *sphyraena* may be an incipient species given its nested phylogenetic position that otherwise renders *chapalae* sensu stricto as paraphyletic (see also multivariate analyses in Fig. 2a, b, Additional file 2: Figs. S2, S4; and pairwise *F*_ST_ analyses in Additional file 3: Tables S4–S7). Until further evidence becomes available (e.g., ecological, ethological), the *sphyraena* group and the sub-species of *C.* *e.* *copandaro* should not be considered species per se as their ancestral polymorphisms have not been fully sorted by genetic drift. Thus, by taking into account multiple lines of evidence (population genomics, phylogenomics, morphology, biogeography, and ecology), we propose that *estor*, *humboldtianum,* and *chapalae* groups constitute three well-differentiated species (*C.* *estor*, *C.* *humboldtianum* sensu stricto, and *C.* *chapalae*, respectively).

The observed discordance between our genomic analyses, which resolved individuals from each lake as monophyletic, and previous studies [11, 18] where clades are not so cohesively clustered by geography, suggests that few mitochondrial and nuclear markers do not have sufficient statistical power to resolve the phylogenetic relationships of this recently diverged group. Here, we show that the use of thousands of SNP loci collectively provide strong power to detect phylogenetic signal while reducing the probability of stochastic error [53], as has also been demonstrated in several species of freshwater [46, 54–57] and marine [58–61] fishes. We observe a discordance between the phylogenetic position of the three main clades, where the *mt*DNA tree places individuals from the *estor* group as early branches while the ddRADseq analyses place them in a more nested position. Such disagreement could be related to the characteristics of the type of markers (e.g., matrilineal inheritance). Mitonuclear discordance can lead to an inaccurate estimation of the evolutionary history of species, ultimately misguiding species delimitations [62, 63, 64]. Unlike the *mt*DNA trees, the ddRADseq analyses align with the geography and geology of central Mexico, identifying geographically cohesive clades.

Finally, in all cases, the nine-species model was strongly rejected thus refuting the morphological-based hypothesis sensu Barbour [10]. Our study represents a rare case where genome-wide data evidences an over estimation of species diversity based on morphological characters. The delineation of such morphospecies was based on several characters, particularly head and body traits, that have been considered as the basis for the current taxonomy in the genus. However, trait measurements such as jaw length, jaw shape, teeth size, and body shape are subjected to great environmental plasticity related to the species’ trophic ecology and habitat characteristics, making it difficult to find diagnostic characters among *Chirostoma* species [10, 19, 65].

### Selection across lakes

The study of alleles involved in local adaptation can unveil loci responsible for adaptive differences among populations. Our analyses of outlier loci detected 294 putative loci under selection, of which at least 106 are related to important biological processes (Additional file 3: Table S4). For example, we detected two SNPs associated with immune response loci (*kpna3* and *bcl11b* genes). Previous studies in trout [67] and sticklebacks [68–70] have highlighted the importance of these loci in the response to different pathogens. Thus, it is possible that the selection of loci associated with the immune response could be related to exposure to lake-specific pathogens during the colonization of new habitats, promoting the local adaptation and divergence of the *humboltianum* group once geographic isolation started.

Other genes detected in this analysis were the *or132-1*, which is an odor receptor associated with the detection of food in the aquatic system [71], and *sws2a*, *lws2a*, and *rh2-1* associated with vision. These findings could be related to the photic environment of each lake: whereas Lakes Chapala and Pátzcuaro are shallow eutrophic water bodies with a high sediment charge of turbid water [72, 73], Lake Zacapu is a clear-water lake where the light reaches an average depth of 4.3 m (up to 11.5 m in some places; [13]). Differences in photic conditions can affect the planktonic community within each lake, promoting differential selection as related to olfactory and visual receptors [74]. Finally, loci associated with the skeleton-muscle apparatus, such as nebulin (*neb*) and titin (*ttn*), and genes involved in growth hormone regulation, such as cullin-associated NEDD8-dissociated protein 1 (*cand1*), and a small glutamine-rich tetratricopeptide repeated containing alpha (*sgta*), could also be involved in the phenotypic changes associated with body size.

We note that these inferences need to be taken with caution as there is a limited performance of *F*_ST_ outlier approaches in non-model organisms to identify candidate genes without a reference genome [75], particularly if the demographic history of the species is not modeled accurately [76]. Reduced representation genomic libraries that use restriction enzymes to cut the DNA may fail to identify key loci as these techniques only capture a small portion of the genome [77, 78], while many loci are lost due to low coverage and filtering [66, 79]. Without a reference genome, these results are highly sensitive to false positives, such as loci linked to sites experiencing purifying selection that can be confounded with processes of local adaptation [66]. Thus, the set of candidate genes identified in this study provides a starting point for further targeted research into underlying genetic bases of selection in the *humboltianum* group.

### Genomic diversity, fishery management and conservation

Our genome-wide SNP analyses revealed that genomic diversity within the *C.* *humboldtianum* species complex is low (Table 2), a pattern that has been also reported in other lacustrine fish species such as the Nile tilapia [80] and sticklebacks [69]. The low heterozygosity in freshwater fish species may be related to smaller effective population sizes and varying demographic histories involving bottlenecks during the recent colonization of new freshwater habitats [69]. The *humboldtianum* group diverged and colonized the lakes of the central Mexico plateau during recent evolutionary times, between 76 and 580 thousands of years [11]. Such sudden demographic expansion from a smaller number of founders [81] may explain their low genetic diversity. In addition, these fishes have recently decreased demographically due to over-exploitation by commercial fisheries, which could have also influenced their genomic diversity [82]. Although the majority of the results of *LDNe* and *HetExcessNe* estimators produced infinite values indicating the effect of sampling error caused by the small sample size (1–24) [83, 84], the *Ne* values observed for the *humboltianum* group were similar to or even smaller than those observed in other lacustrine fish species (e.g., *Amphilophus* *labiatus* and *A.* *citrinellus* [85]), or species that have experienced a population collapse (e.g., *Oscorhynchus* *nerka* [86]).

Although we have no evidence of the presence of hybrid individuals in natural lakes, as has been previously reported [10, 87], given the frequent translocation events promoted by aquaculture policies since 1970 [88, 89], we detected some hybrid individuals of *C.* *humboldtianum* sensu stricto in artificial dams that are genomically closer to the clade I. These hybrids could be the result of deliberate introductions of different species of the “*humboltianum* group” into several artificial dams of the region [8, 81, 88], which were done to promote artisanal fisheries of this important resource and to improve the local economy. Future studies should evaluate the effectiveness of the introductions in the local fisheries and their impact on natural populations within the *humboltianum* group. Some hybrid individuals may show hybrid vigor and could become better competitors than local species, making this practice another factor affecting natural populations [8].

*Chirostoma* species represent a highly important economic and cultural resource since pre-Hispanic times [8]. However, species in this genus have been heavily overfished, leading to the collapse of several populations and severe conservation problems where some species are now considered extinct or in danger of extinction across several locations [8, 9]. Currently, the *humboldtianum* group is threatened by several factors, particularly habitat loss, pollution, the introduction of exotic species, and overfishing [50]. The decrease of silverside fish populations has also caused the collapse of their fisheries, negatively impacting the local fishermen’s communities [8]. Another consequence of the over-exploitation of these fishes is the decrease in the capture size and the age of maturity size [8, 9]. Thus, the delimitation of operational genomic units is critical for fisheries management and conservation plans [8]. Additionally, information on the genomic structure and genetic diversity within and between natural populations of the *humboldtianum* group are crucial to understanding their ability to cope with environmental changes over evolutionary time [90]. Our results support the presence of four genomic groups within the *humboltianum* group, distributed in the Lakes Chapala, Pátzcuaro-Zirahuén, and Zacapu. We strongly recommend revising management and conservation plans taking into consideration our proposed species boundaries.

## Conclusions

Our genome-wide analyses based on ~ 2 K to  ~ 33 K SNP loci, examined in light of morphological and ecological lines of evidence, provides remarkable resolution to address a convoluted case of species delimitation within the *humboldtianum* group, a result not previously achieved with the use of a small number of *mt*DNA and *n*DNA markers. We resolved four genomic clusters arranged into three geographically cohesive clades (clade I, *chapalae*, and *sphyraena* groups from Lake Chapala; clade II, *humboldtianum* sensu stricto group from Lake Zacapu and dams; and clade III, *estor* group from Lakes Pátzcuaro and Zirahuén). These groups conflict with the previously described morphospecies, and also with the ‘peces blancos’ and ‘charales’ ecotypes. Notably, our genomic analyses reject both morphology- and ecotype-based delineations.

Our results suggest that the main cladogenetic events that gave rise to the three clades within the *humboldtianum* group resulted from allopatric processes generated by the complex geologic history of the Lerma-Chapala paleo system, while intra-lake divergence of the *sphyraena* group could be the product of ecological and incipient speciation. It is important to highlight that lumping the nine morphospecies into three does not imply reducing conservation efforts but enforcing the inclusion of molecular information to create management strategies and conservation plans. Critically, the low levels of genetic diversity and *Ne* values observed inside each genomic cluster should be considered in any further conservation efforts. All in all, our study represents a rare case where the use of genome-wide data evidence taxonomic over-splitting based on morphological information, while it emphasizes the use of approaches that take into account multiple lines of evidence to address complex species delimitation scenarios.

## Methods

### Sample collections

Our research is a follow-up study of a recently published phylogeographic analysis of the *humboldtianum* group based on two mitochondrial genes (cytochrome b [*Cytb*], and a fragment of the hypervariable control region [*D-loop*]), and one nuclear locus (first intron of the S7 ribosomal protein gene [*S7*]) [11]. We carefully selected 77 individuals representing the nine nominal species of the *humboldtianum* group and the genetic diversity observed by Betancourt-Resendes et al. [11] to build a genomic dataset. We added two individuals of *Chirostoma* *jordani*, and one of *Chirostoma* *attenuatum* as outgroups. We collected the samples from local fishermen bycatch in 2014–2018, following the ethical capture methods and regulations approved by the Official Mexican Norm NOM-032-SAG/PESC-2015 and NOM-036-SAG/PESC-2015 for fishing in the lakes of central Mexico. Fishes were euthanized by applying rapid chilling protocols that consisted in placing them in ice water-cold baths (2–4 °C), as recommended by the American Veterinary Medical Association (AVMA; https://www.avma.org/resources-tools/avma-policies/avma-guidelines-euthanasia-animals). Voucher specimens were fixed in 10% formaldehyde and preserved in 70% ethanol. Our sampling spans six localities, which together cover the distribution range of the *humboldtianum* group in the central Mexico plateau (Additional file 3: Table S1; Fig. 1). We sampled fin clips, preserved them in 95% ethanol, and stored them at − 76 °C. We deposited tissue and voucher at the fish collection of the Universidad Michoacana de San Nicolás de Hidalgo (UMSNH), Mexico (Additional file 1: Appendix 1). None of our experiments included living organisms.

### Molecular protocols, de novo assembly, and SNP genotyping

We extracted high-molecular-weight DNA using the Qiagen DNeasy Blood and Tissue kit (Qiagen, Inc.) following the manufacturer’s protocol. We prepared the ddRAD sequencing libraries at the Sequencing and Genotyping Facility (SGF) at the University of Puerto Rico-Río Piedras (UPR-RP), following the protocol of Peterson et al. [91]. We used the *PstI* and *MseI* restriction enzymes with a size selection window of 300–600 bp. We sequenced ddRADseq libraries in two lanes of Illumina HiSeq 4000 PE 100 bp at the Knapp Center of Biomedical Discovery (KCBD) Genomics Facility, University of Chicago.

We verified the quality of the raw reads using the software FastQC v0.11.5 (http://www.bioinformatics.babraham.ac.uk/projects/fastqc/). We demultiplexed the sequenced libraries and removed the restriction sites using the process_radtags.pl script implemented in Stacks v2.4 [92, 93]. We trimmed all demultiplexed reads to 86 bp after removal of the restriction site overhangs. We then applied a second quality filter using a Phred score of 33, producing a total of 3.5 × 10^8^ reads that we retained. We deposited all demultiplexed raw reads in GenBank (NCBI) (accession no. SAMN26725252–SAMN26725331, SRA BioProject PRJNA816865).

As there is no *Chirostoma* genome that we could use as a reference, we conducted a de novo assembly of putative loci using Stacks. To select the assembly parameters that best-fit the data, we first performed an exploratory analysis using default parameters. We then used a subset of 15 samples, formed by the individuals of each nominal species with the highest coverage to conduct a second assembly by applying different combinations of parameters as reported by Mastretta-Yanes et al. [94], Paris et al. [95], and Pedraza-Marrón et al. [96]. Details of the protocol for the de novo assembly are given in the Supplementary Material (SM, Additional file 2: Figs. S11–S13). We conducted the final locus assembly using a minimum of five raw reads required to form a stack (*m* = 5), with a maximum of four mismatches between stacks (*M* = 4), and five mismatches between loci of different individuals (*n* = 5).

### SNP filtering and database selection

SNP filtering parameters play an important role in the number of recovered loci and the inferred degree of genetic differentiation [97, 98]. We thus implemented a series of exhaustive steps to select the final SNP loci that were included in further analyses (Additional file 2: Fig. S14). (*Step* *1*) We filtered biallelic loci according to the number of individuals, populations, and nominal species (Additional file 3: Table S2). We selected four databases that ranged between ~ 1000 and ~ 105,000 SNPs to apply further filters. (*Step* *2*) We removed low-frequency alleles and potential paralogous loci using a minor allele frequency (*maf*) of 0.01 and 0.05. (*Step* *3*) We selected sites with different tolerance thresholds for missing data (0.05, 0.25, 0.50, and 0.75). (*Step* *4*) We removed individuals with various cutoffs of missing data (0.05–0.99). Collectively, these filters produced 24 additional datasets, with 150–33,800 SNP loci, 0.3–49% of missing data, and 37–80 individuals representing 4–9 morphospecies. (*Step* *5*, Additional file 3: Table S3). We then selected 19 (out of the 24) matrices to assess the robustness of our analyses to differences in numbers of individuals (37–72), nominal species (4–9), SNPs (~ 2 k– ~ 33 k), and proportions of missing data (0.3–49%). The matrices generated during this step were further used to evaluate the sensitivity of the analyses to the exclusion of nominal species with high levels of missing data (see SM). For more exhaustive analyses, we also selected five (out of the 24) datasets with a final set of 72 individuals representing the nine morphospecies: A-33716snps (33,716 SNPs, 15.7% missing data), B-10517snps (10,517 SNPs, 7.7% missing data), C-4821snps (4821 SNPs, 12.3% missing data), D-3564snps (3564 SNPs, 10.4% missing data), and E-1887snps (1887 SNPs, 12.4% missing data). (*Step* *6*) Finally, to conduct *F*_ST_ outlier analyses (see below), the five matrices (A–E) from previous step were partitioned into all, neutral, and outlier loci, for a total of 15 databases. For details refer to the SM (see also Additional file 2: Fig. S14).

### Multivariate analyses

To determine the number of genetic clusters within the *humboldtianum* group, we conducted a principal component analysis (PCA)—designed to identify genetic groups through eigenvector decomposition of allele frequencies [99]—using matrices A–E with all, neutral, and outlier SNP loci. In addition, to identify de novo structure, we conducted an assessment of a priori designations using a discriminant analysis of principal components (DAPC). DAPC combines discriminant (DA) with principal component (PC) analyses to maximize genetic variance among groups while minimizing within-group variance [100, 101]. We selected the a priori groups by configuring the nine nominal *Chirostoma* morphospecies. We assessed the most likely number of clusters (*k* = 1–9) using the *find.clusters* function within the *adegenet* v2.1.1. package [102] by selecting the *k* model with the lowest Bayesian Information Criterion (BIC) score. We calculated the number of PCs to be retained using the *dapcCross* validation function, which is based on the highest successful assignment that presented the lowest mean squared error [100].

### Genomic structure and genomic differentiation analyses

To evaluate the number of genetic clusters within the *humboldtianum* group, we analyzed the final matrices (A–E) with all, neutral, and outlier loci under a maximum-likelihood framework using the software ADMIXTURE v1.3.0 [103]. This program estimates the proportion of the ancestral population (*Q* estimates) for each individual to calculate the number of genetic clusters (*k*) under a cross-validation procedure, where lower values represent the optimal number of populations [104]. All analyses were run with *k* values ranging from 1 to 12, which represent the nine nominal morphospecies described for the group, the sub-species from Zirahuén Lake, *C.* *e.* *copandaro*, and two additional genomic groups that could be potentially be detected if a higher number of species exist. To further assess population clustering, we conducted admixture analyses within Chapala and Pátzcuaro-Zirahuén lakes, corresponding to clade I and clade III, respectively (see Results). The analysis for Chapala lake sample (n = 41) was run with *k* values ranging from 1 to 5, which represent the five morphospecies of *humboltianum* group described from this lake. The analysis for Pátzcuaro-Zirahuén lakes sample (n = 24) was run with *k* values ranging from 1 to 4, which represent the three morphospecies of *humboltianum* group from Pátzcuaro plus the subspecies *C.* *e.* *copandaro* from Zirahuén. We plotted all cross-validation and *Q* estimates using the *ggplot2* [105] and *pophelper* [106] packages in R studio.

We computed analyses of pairwise *F*_ST_ comparisons to test the genetic differentiation among groups using the D-3564snps-all_loci matrix. We selected this matrix as it showed the general patterns observed with other matrices, but also because it had the highest percentage of variation explained obtained with multivariate analyses (see “Results”). We examined four alternative species delimitation schemes: (i) nine morphospecies (morphological-based hypothesis; sensu Barbour [10]), (ii) five mitonuclear groups (*mt*DNA/nDNA-based hypothesis; sensu Betancourt-Resendes et al. [11]), (iii) four genomic clusters (*k* = 4) observed with the DAPC analyses (see “Results”), and (iv) three clusters detected by the admixture analyses (*k* = 3) (see “Results”). To further test whether an agreement between species’ body size and trophic speciation exists [10], we calculated pairwise *F*_ST_ comparisons among ecotypes by lake (‘peces blancos’ vs*.* ‘charales’). Details on ecotype discrimination are provided in the SM. We calculated these analyses in ARLEQUIN v3.5.1.2 [107], and significance was determined using 10,000 permutations and a Bonferroni correction [108] to adjust *p* values for these calculations (significant levels of α are provided in Additional file 3: Tables S4–S8).

### Phylogenetic analyses

We estimated phylogenetic trees under a maximum likelihood (ML) framework using the software IQ-TREE v2.0.6 [109]. We conducted phylogenetic inference for the five matrices (A–E) including all, neutral, and outlier SNP loci, and using *C.* *jordani* as the outgroup. All analyses used the GTR model with gamma distribution and the ascertainment bias correction (ASC) to account for acquisition discrepancies related to SNP datasets. We estimated branch support using IQ-TREE’s ultrafast bootstrap algorithm (UFBoot) with 500 replicates [110].

To evaluate patterns of incongruent evolutionary histories between mitochondrial and nuclear loci—mitonuclear discordance—we used the sequence data of two mitochondrial genes (cytochrome b or *Cytb*, and a fragment of the hypervariable control region, *D-loop*) from Betancourt-Resendes et al. [11]. Using the GTR model in IQ-TREE as explained above, we estimated ML *mt*DNA trees for each gene separately and also for the concatenated *mt*DNA matrix. We extracted tips shared between the *mt*DNA trees and our ddRADseq (D-3482snps-neutral_loci matrix) using the *keep.tip* function implemented in the R package *phytools* [111].

### Multispecies coalescent analyses

We inferred a multi-coalescent species tree for the D-3482snps-neutral_loci matrix using the SNAPP v1.3.0 plug-in [112] implemented in BEAST2 v2.6.3 [113]. SNAPP allows the inference of a species tree from unlinked SNP data while bypassing gene tree inference [112]. For this analysis we used two chains of 30 million steps, sampling every 500 trees, with a burn-in of 10%. We set default priors for coalescent and mutation rates, as well as ancestral population size parameters. We visualized all results in the software Tracer v1.7 [114] to confirm that the analyses had converged, reached stationary, and that all effective sample sizes (ESS) were higher than 200. Lastly, we summarized the conflict of posterior distribution trees into the species tree using DENSITREE v2.01 [115].

To test the previously outlined species delimitation scenarios (i–iv) in a coalescent framework, we applied the Bayes Factor Delimitation (BFD*) approach [116] using SNAPP and BEAST2. To overcome computational burden, we generated three subsets encompassing the nine morphospecies with 39–59 individuals and 411–1102 SNP loci that were generated from the D-3482snps-neutral_loci matrix (see Additional files). With BFD* candidate species delimitation scenarios are evaluated according to the marginal likelihood estimates (MLE). The different scenarios are then compared using Bayes Factors (BF) [117], estimated by subtracting MLE values for two models and multiplying the difference by two (BF = 2 × (MLE_1_ − MLE_2_)). As only two models are compared at the time, we evaluated all possible combinations among the species delimitation scenarios (see Table 1; Additional files). To set up the priors and MCMC runs, we followed the recommendations provided in the BFD* manual [118]. The speciation rate (λ), which represents the birth-rate on the Yule tree prior, was set to follow a gamma distribution with α = 2 and β = 200.

### Genetic diversity, effective population size (*Ne*), and *FST* outlier analyses

We estimated the genomic diversity of the *humboldtianum* group considering the four species delimitation scenarios outlined above. We did not test a scheme that included ecotypes as we did not find any support for this hypothesis (see Results). We used GenAlEx v6.5 software [119] to calculate the observed (*Ho*) and expected (*He*) heterozygosity for each cluster using D-3482snps-neutral_loci matrix. Additionally, we evaluated the effective population size (*Ne*) of each species delimitation scenario by estimating linkage disequilibrium [83], heterozygote excess [120], and molecular co-ancestry values [121] with the software NeEstimator v2 [122].

To detect loci with high levels of genetic differentiation, we examined the A–E matrices formed by all SNP loci under a Bayesian framework using the program BayeScan [123]. In this analysis, *F*_ST_ coefficients are decomposed into a population-specific component (β) shared by all loci, and a locus-specific component (α) shared by all populations. When a locus-specific component is necessary to explain the data, selection is assumed to play a role at that locus [123]. To reduce the risk of false positives without reducing the power to detect loci evolving under selection, we set the default parameters and priors for a neutral model according to the total number of SNP loci in each matrix (100 for matrices with > 1000 loci, and 1000 for matrices with > 10,000 loci) [124]. Posterior Odds (PO) values were used to detect loci under section in the context of multiple testing. PO are simply the ratio of posterior probabilities and indicate how more likely the model with selection is compared to the neutral model (Jeffrey’s scale of evidence can also be used with posterior odds). A big advantage of PO values over posterior probabilities is that they directly allow the control of the False Discovery Rate [124]. Statistical significance for outlier loci was assumed if *q* values ≤ 0.05. To determine the strength and direction of selection, we estimated the α parameter, where a positive value suggests diversifying selection and a negative value indicates balancing or purifying selection [124]. Lastly, to identify the approximate genomic region within which the outlier loci occur, we searched the consensus stack sequences on the NCBI server (https://blast.ncbi.nlm.nih.gov/Blast.cgi) using the Blastn algorithm. To choose the most similar candidate sequence, we used an e-value threshold lower than 0.05, a maximum target sequence of 5000 to retain all possible hits, and a sequence similarity above 75% to filter among the retained sequences. We ran all R analyses using R studio v1.2.1335 and R v4.1.1 [125].

## Supplementary Information

**Additional file 1: Appendix S1.** Samples included in this study indicated the groups to which they belong in each of the different species hypotheses, analyses results, ecotypes, locality, and lake.

**Additional file 2:** Supporting Methods. **Figure S1.** Principal Components Analyses 19 matrices with different numbers of SNPs loci, missing data, individuals, and species of the *humboldtianum* group. **Figure S2.** Principal component analyses (PCAs) based on all, neutral, and outlier SNP loci from matrices A, B, C, and E. PCAs estimated using all and neutral SNPs (~ 2 k–33 k) consistently recovered four genomic groups that are in agreement with geographic patterns but not with the previously recognized morphospecies; these are delimited with convex hulls: *humboldtianum* sensu stricto group (blue), from Lake Zacapu; *estor* group (green) from Lakes Patzcuaro and Zirahuén; *chapalae* group (red), from Lake Chapala; and *C.* *sphyraena* group (yellow), also from Lake Chapala. PCA analyses based on ~ 100–400 SNPs also resolved four genomic groups in matrices A and B, and in matrices C and E, *chapalae* and *sphyraena* groups clustered together. Morphospecies are color-coded according to the genomic groups observed. **Figure S3.** Plots of Bayesian Information Criterion (BIC) *vs.* number of clusters (*k*) of DAPC analyses for all, neutral, and outlier SNP loci from matrices A–E. **Figure S4.** Discriminant analyses of principal components (DAPCs) estimated using *ca.* 1800–33,700 SNPs resolve four well-differentiated clusters. DAPCs based on outlier SNPs recover three to four groups. These results are largely consistent across analyses based on matrices A, B, C, and E (a–d), and are also concordant with PCA analyses. In neither case, the observed genomic clusters do correspond to the morphology-based species delimitation scenario. Morphospecies are color-coded according to the genomic clusters recovered: blue, *humboldtianum* sensu stricto, green, *estor* group; red, *chapalae* group; yellow, *C.* *sphyraena*. **Figure S5.** Plots of cross-validation error of each *k* (number of clusters) analyzed in Admixture analyses for all, neutral, and outlier SNP loci from matrices A- E. The cross-validation procedure was performed with the folds value = 5 (the default), a block relaxation algorithm as point estimation method, and the point estimation terminated with the objective function delta < 0.0001. **Figure S6.** Admixture assignment analyses estimated using *ca.* 80–33,700 neutral and outlier SNPs consistently identified three well-differentiated clusters (*k* = 3) using matrices A, B, C, and E (a–d). Outlier SNPs show an increased intermingling of individuals among clusters compared with analyses based on all or neutral loci. Each bar represents the probability of assignment to each cluster. Genomic clusters are color-coded as blue, *humboldtianum* sensu stricto, green, *estor* group; orange, *chapalae-sphyraena* group. CHA, Lake Chapala; TEP, Tepuxtepec Dam; TRI, Trinidad Fabela Dam; PAT, Lake Pátzcuaro; ZIR, Lake Zirahuén. **Figure S7.** Admixture assignment analyses estimated using *ca.* 33,700 SNP loci a) in Chapala Lake, and b) within Lakes Pátzcuaro-Zirahuén. **Figure S8.** Phylogenetic trees of *ca.* 1800–33,700 SNP loci of the *humboldtianum* group estimated under a maximum likelihood framework in IQ-TREE. Phylogenetic trees were estimated using all (~ 1900–33,700), neutral-only (~ 1800–33,300), and outlier (~ 80–350) SNPs. Individuals are color-coded by genetic clusters according to Fig. 2. **Figure S9.** Mitochondrial trees (left) and ddRADseq phylogeny (right) of the *humboldtianum* group. The tips in the RADseq inferences were pruned to include the same individuals present in the mitochondrial trees. **Figure S10.** *F*_ST_ *versus* log10-transformed posterior odds (PO) values for the global outlier detection calculated in BayeScan. The analyses were estimated considering the nine morphospecies and (A) 33,716 SNPs, where the vertical line represent the FDR threshold of *q* = 0.037; (B) 10,157 SNPs, *q* = 0.040; (C) 4821 SNPs, *q* = 0.025; (D) 3564 SNPs, *q* = 0.034; and (E) 1887 SNPs, *q* = 0.015. **Figure S11.** Mean coverage of the subset with15 samples using different values of the minimum raw reads required to form a stack (*m*1–*m*6). **Figure S12.** Putative loci at different combinations of de novo assembly parameters: *m*, minimum reads required to form a stack; *M*, allowed SNPs in a stack required to form a putative locus in an individual; *n*, allowed SNPs in a stack required to form a locus in the population. Each parameter was changed one at the time (*m* = 2–6, *M* = 0–6, and *n* = 0–11) while keeping the others at default values (*m*3*M*2*n*1). **Figure S13.** Genotyped loci and variant sites using a constraint on the number of minimum individuals in a population required to have that locus (r = 40, 60, and 80) based on different cutoff, as follow: (A) minimum raw reads required to form a stack (*m* = 2–6), (B) maximum mismatches allowed between stacks of the same individual (*M* = 0–6), and (C) mismatches allowed between loci of different individuals (*n* = 0–11). **Figure S14.** Flow chart of the quality control and SNP filtering steps applied to generate the final datasets.

**Additional file 3: Table S1.** Information of the sampling localities in the central Mexico plateau: localities, lakes, number of samples (n), geographic coordinates (Latitude and Longitude) and Altitude (meters over sea level, m.o.s.l.). **Table S2.** Filters applied to SNP databases. The databases highlighted in bold were selected for further filtering. **Table S3.** Databases generated with Tassel filters used to evaluate the consistency of the genomic patterns to the number of SNPs, individuals, species, and missing data percentage. The matrices used in the genomic analyzes are indicated in bold. **Table S4.** Genomic pairwise *F*_*ST*_ comparisons for 3482 SNP loci among the nine morphospecies proposed by Barbour [10]. Values in bold indicate significance at α = 0.0014 for pairwise comparisons, following sequential Bonferroni corrections. **Table S5.** Genomic pairwise *F*_*ST*_ comparisons for 3482 SNP loci among the five mitonuclear groups proposed by Betancourt-Resendes et al. [11]. Values in bold indicate significance at α = 0.005 for pairwise comparisons, following sequential Bonferroni corrections. **Table S6.** Genomic pairwise *F*_ST_ comparisons for 3842 SNP loci among the four genomic clusters detected in *humboldtianum* group by DAPC analyses. Values in bold indicate significance at α = 0.0083 for pairwise comparisons, following sequential Bonferroni corrections. **Table S7.** Genomic pairwise *F*_ST_ comparisons for 3482 SNP loci among the three genomic clusters detected in the *humboldtianum* group by Admixture and phylogenetic analyses. Values in bold indicate significance at α = 0.017 for pairwise comparisons, following sequential Bonferroni corrections. **Table S8.** Genomic pairwise *F*_ST_ comparisons for 3482 SNP loci among the ecotypes in each lake. Values in bold indicate significance at α = 0.005 for pairwise comparisons, following sequential Bonferroni corrections. **Table S9.** Outlier loci detected by the *F*_ST_ outlier analysis performed in BayeScan related to gene regions.

## Data Availability

Raw sequence data have been deposited in the NCBI SRA BioProject no. PRJNA816865 (accession no. SAMN26725252–SAMN26725331). SNPs genomic matrices are available from the Dryad Digital Repository https://doi.org/10.5061/dryad.w0vt4b8rz. All corresponding sample codes and information are included in Additional file 1: Appendix 1.
