# Supplementary material for: Genome-wide species delimitation analyses of a silverside fish species complex in central Mexico indicate taxonomic over-splitting
Source: BMC Ecol Evol. 2022 Sep 14;22:108. doi: 10.1186/s12862-022-02063-0 (PMC9472351; doi:10.1186/s12862-022-02063-0)
Supplement: Supplementary file 2 — Additional file 2: Supporting Methods. Figure S1. Principal Components Analyses 19 matrices with different numbers of SNPs loci, missing data, individuals, and species of the humboldtianum group. Figure S2. Principal component analyses (PCAs) based on all, neutral, and outlier SNP loci from matrices A, B, C, and E. PCAs estimated using all and neutral SNPs (~ 2 k–33 k) consistently recovered four genomic groups that are in agreement with geographic patterns but not with the previously recognized morphospecies; these are delimited with convex hulls: humboldtianum sensu stricto group (blue), from Lake Zacapu; estor group (green) from Lakes Patzcuaro and Zirahuén; chapalae group (red), from Lake Chapala; and C. sphyraena group (yellow), also from Lake Chapala. PCA analyses based on ~ 100–400 SNPs also resolved four genomic groups in matrices A and B, and in matrices C and E, chapalae and sphyraena groups clustered together. Morphospecies are color-coded according to the genomic groups observed. Figure S3. Plots of Bayesian Information Criterion (BIC) vs. number of clusters (k) of DAPC analyses for all, neutral, and outlier SNP loci from matrices A–E. Figure S4. Discriminant analyses of principal components (DAPCs) estimated using ca. 1800–33,700 SNPs resolve four well-differentiated clusters. DAPCs based on outlier SNPs recover three to four groups. These results are largely consistent across analyses based on matrices A, B, C, and E (a–d), and are also concordant with PCA analyses. In neither case, the observed genomic clusters do correspond to the morphology-based species delimitation scenario. Morphospecies are color-coded according to the genomic clusters recovered: blue, humboldtianum sensu stricto, green, estor group; red, chapalae group; yellow, C. sphyraena. Figure S5. Plots of cross-validation error of each k (number of clusters) analyzed in Admixture analyses for all, neutral, and outlier SNP loci from matrices A- E. The cross-validation procedure was perform [file 12862_2022_2063_MOESM2_ESM.docx]

**Additional File 2**

**Genome-wide species delimitation analyses of a silverside fish species complex in central Mexico indicate taxonomic over-splitting**

Victor Julio Piñeros, Carmen del R. Pedraza-Marrón, Isaí Betancourt-Resendes, Nancy Calderón-Cortés, Ricardo Betancur-R and Omar Domínguez Domínguez

**Supporting Methods**

**Supplementary Figures**

**Supplementary References**

**Supporting Methods:**

**Molecular protocols and SNP genotyping.** We extracted DNA from fin clips of 77 individuals of the nine nominal species of the *humboldtianum* group (*sensu* Barbour 1973), using the Qiagen DNeasy Blood and Tissue kit (Qiagen, Inc.) following the manufacturer's protocol. We also included two specimens of *Chirostoma jordani* and one of *Chirostoma attenuatum* as outgroups. We prepared the ddRADseq libraries at the Sequencing and Genotyping Facility (SGF) at the University of Puerto Rico - Río Piedras (UPR-RP) using the protocol of Peterson *et al.* (2012). To this end, we used the restriction enzymes *PstI* and *MseI*, and a size selection window of 300–600 bp. We included 20 individuals per library using a set of six base-pairs barcodes in combinatorial schemes to index each individual. We sequenced the resulting libraries in one Illumina HiSeq 4000 lane using 100 base pair-ended sequencing at the Knapp Center of Biomedical Discovery (KCBD) Genomics Facility at the University of Chicago.

We checked the quality of a total of 358,591,878 raw reads with FASTQ 0.11.5 (www.bioinformatics.babraham.ac.uk/projects/fastqc). We used the *process_radtags* pipeline available in Stacks v2.4 [3, 4] to demultiplex our ddRADseq libraries. We applied a quality control Phred score of 33 to filter demultiplexed reads and trimmed the sequences to 86 bp after removing the enzyme's overhangs. We used a total of 349,891,799 reads that passed the filters (97.6%) to assemble putative loci.

We conducted a *de novo* assembly pilot run using *denovo.pl* program and default parameters in Stacks on all 80 individuals. As the selection of key parameters for *de novo* assembly (*m* = minimum raw reads required to form a stack, *M* = maximum mismatches between stacks, and *n* = mismatches between loci of different individuals) greatly influences the quality and formation of putative loci [5], we selected a subset of 15 samples (including individuals of each nominal species that presented the highest coverage values) to optimize the assembly parameters that best fit our data. We followed a combination of the protocols used by Mastretta-Yanes *et al.* (2015), Paris *et al.* (2017), and Del Pedraza-Marrón *et al.* (2019), by varying one parameter at the time (*m* = 2–6, *M* = 0–6, and *n* = 0–11). We observed a common pattern of RADseq data in which higher values of *m* increased the average sample coverage (Figure S1) but decreased the number of putative loci (Figure S2). Overall, increasing *m* from three to six produced a lower number of putative loci. Based on these results, we selected a value of five, as higher values could exclude true alleles, underestimating the number of heterozygous loci in the dataset [6]. Finally, we assessed the variation of putative loci while constraining the selection to genetic information available of the individuals in a population (*r* =40, 60, and 80) (Figure S3). After a maximum of four mismatches between stacks (*M* = 4) and five mismatches between loci of different individuals (*n* = 5), the number of putative loci stops dropping drastically. Therefore, we selected a final combination of *m*5*M*4*n*5 to perform the *de novo* assembly on the 80 individuals.

Given the lack of a reference genome that guided the loci assembly, we conducted a series of quality filter steps to form the final datasets used in further analyses (Figure S4).

*Step 1.* We filtered biallelic loci using Stacks according to the number of populations (*p* = minimum populations), individuals (*r* = minimum percentage of individuals in a population), and samples (*R* = minimum percentage of samples overall), selecting only the first single nucleotide polymorphism (SNP) per tag to avoid linkage between loci. We calculated the percentage of missing data for each dataset (Table S2) with VCFtools v0.1.15 [8], after which we selected four databases (pop_r80, pop10_r80, R80, and R85; Table S2) that included between ~1000 and ~105000 SNPs loci and 9.3 - 48.9% of missing data.

*Step 2.* To exclude low-frequency alleles and potential paralogous loci we removed sites with a minor allele frequency (*maf*) of 0.01 and 0.05. We created databases with two different *maf* thresholds as the selected cutoff can affect the population structure estimated by model-based (*e.g.*, admixture) or multivariate approaches (*e.g.*, PCA) [9].

*Step 3.* To remove sites with different tolerance for missing data we applied the ‘min. sites’ filter (0.05, 0.25, 0.50, and 0.75).

*Step 4*. We implemented the taxa ‘min. sites’ filter to remove individuals with different thresholds for missing data (0.05–0.99).

All these filters resulted in 24 datasets ranging among 1,887–33,882 SNP loci, 0.3–48% of missing data, 31–72 individuals, and four to nine species of the *humboldtianum* group (Table S3). All filters described in steps two to four were conducted using the software Tassel 5 v20210210 [10]. We removed five samples (CPUM_35317 and CPUM_35306 of *C. consocium*; CPUM_35300 and CPUM_35305 of *C. lucius*; and CPUM_10617 of *C. estor*) from the analyses because preliminary results showed their genotypes mixed with *C. jordani* (a *Chirostoma* species that does not form part of the *humboldtianum* group), presumably representing hybrid individuals. Additionally, we did not consider the individual of *C. attenuatum* (outgroup) for further analyses due to the bad quality of the genomic data recovered for that individual.

*Step 5*. To test the robustness of our data we conducted preliminary analyses based on 19 databases (Table S3) varying among 37–72 individuals, 4–9 nominal species, and ~2 k to ~37 k SNP loci (Figure S5). Our preliminary results were consistent regarding the number of nominal species included. Hence, we kept the 72 individuals representing the nine morphospecies of the *humboldtianum* group across the sampled localities.

Then we selected five matrices generated with different combinations of *maf* thresholds, missing data (from 7.7–15.77%), and the number of SNP loci (between 1,887 and 33,716 loci), hereafter referred to as A-33716snps, B-10517snps, C-4821snps, D-3564snps, and E-1887snps matrices.

*Step 6*. Finally, to estimate *F*_ST_ outlier analyses (see section 2.7), we separated the five matrices (A–E) by all, neutral-only, and outlier loci for a total of 15 databases that were used in downstream analyses (Figure S4). Neutral-only matrices ranged between 1,795–33,346 SNPs, while the outlier matrices contained 82–370 SNPs.

**Characterization of ecotypes.** *Chirostoma* species in central Mexico have been categorized as ‘peces blancos’ or ‘charales’ ecotypes [1]. We considered the nominal species *C. chapalae*, *C. consocium*, *C. grandocule*, and *C. patzcuaro* as ‘charales’, while *C. sphyraena*, *C. lucius*, *C. promelas*, *C. humboldtianum sensu stricto*, and both subspecies of *C. estor* were categorized as ‘peces blancos’. The main character used to discriminate between both ecotypes is the standard-length SL of the individuals during their adult phase (117–300 mm, 70–142 mm SL, respectively; see also Mercado-Silva *et al.,* 2015). Additional morphological (*e.g.*, jaw length, head length, snout shape, anal fin length, snout pigmentation, and size of the teeth) and meristic (number of pre-dorsal scales, number of lateral-line scales, number of gill rakers) traits are also used for the correct taxonomic identification and discrimination of ecotypes [1, 11]. Herein, all individuals were carefully identified using the morphological diagnostic characters suggested by Betancourt-Resendes *et al.* (2020).

**Bayes factor delimitation analyses (BFD*).** We applied additional filters to the matrix D-3482snps-neutral_loci by retaining individuals from each of the nine morphospecies and low levels of missing data. The subsets assembled were: subset 1, comprising 39 individuals, 411 SNP loci, and 7.2% of missing data; subset 2, 39 individuals, 1102 SNP loci, and 9.1% of missing data; and subset 3, 59 individuals, 548 SNP loci, and 7.9% of missing data. We calculated Bayes factor (BF) values as 2 x (MLE of model 1 - MLE of model 2) and followed the framework provided by Kass & Raftery (1995) to assess the support of the candidate models. Because of the BF comparisons only address two models at the time (model 1 *vs.* an alternative model represented by model 2), we evaluated all possible combinations among the species delimitation scenarios. First, we considered the current taxonomy that includes nine morphospecies as model 1 (model 1 = nine morphospecies *vs.* the alternative models of three, four, and five species). To set up the priors and MCMC runs, we followed the recommendations provided by Leaché and Bouckaert (2018), where we set the birth-rate on the Yule tree prior (λ) to a gamma distribution with α = 2 and β = 200. The XML files used for these analyses are available from xxx.


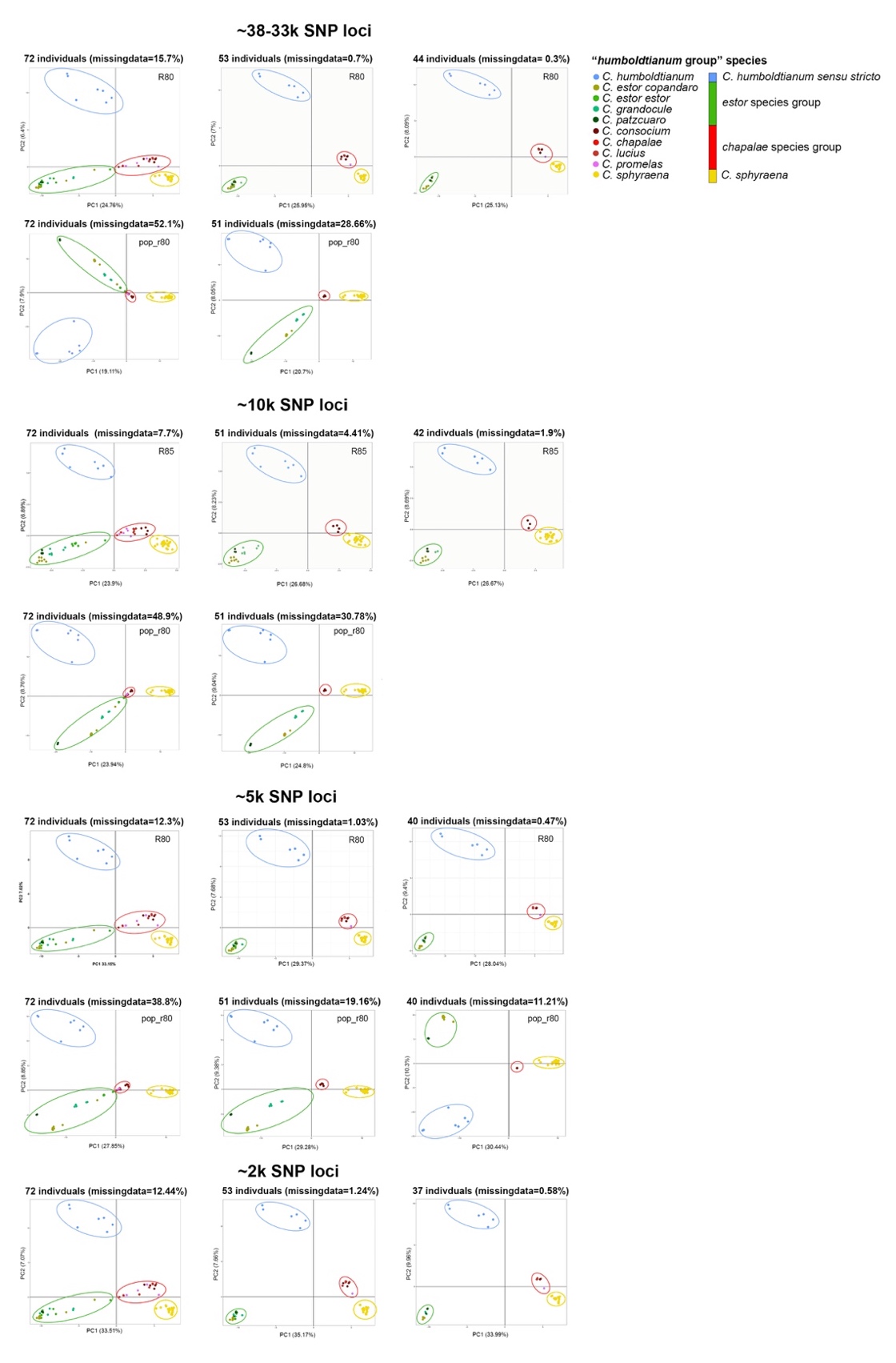


**Figure S1.** Principal Components Analyses 19 matrices with different numbers of SNPs loci, missing data, individuals, and species of the *humboldtianum* group.

**
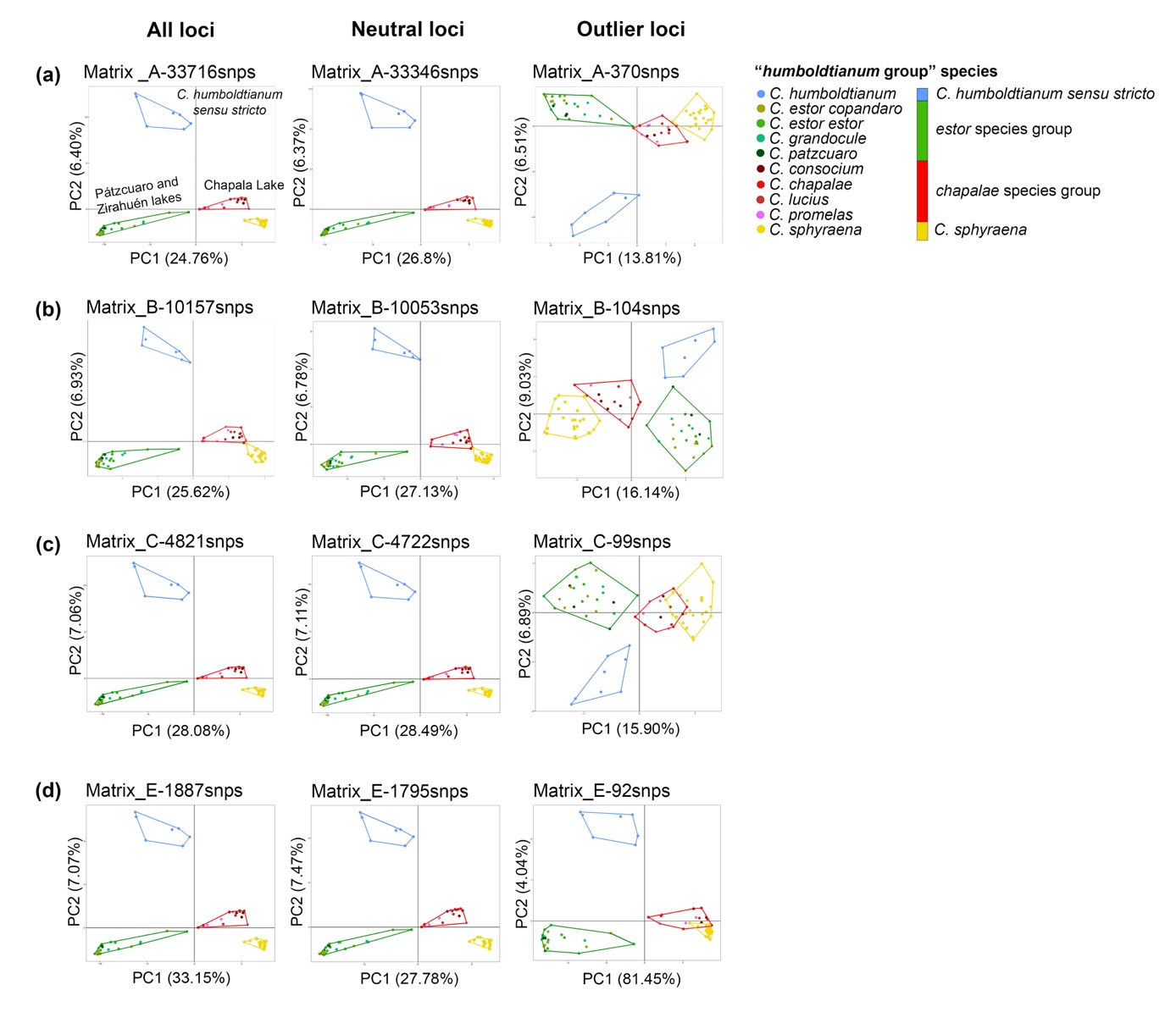
**

**Figure S2.** Principal component analyses (PCAs) based on all, neutral, and outlier SNP loci from matrices A, B, C, and E. PCAs estimated using all and neutral SNPs (~2k–33k) consistently recovered four genomic groups that are in agreement with geographic patterns but not with the previously recognized morphospecies; these are delimited with convex hulls: *humboldtianum sensu stricto* group (blue), from Lake Zacapu; *estor* group (green) from Lakes Patzcuaro and Zirahuén; *chapalae* group (red), from Lake Chapala; and *C.* *sphyraena* group (yellow), also from Lake Chapala. PCA analyses based on ~100–400 SNPs also resolved four genomic groups in matrices A and B, and in matrices C and E, *chapalae* and *sphyraena* groups clustered together. Morphospecies are color-coded according to the genomic groups observed.


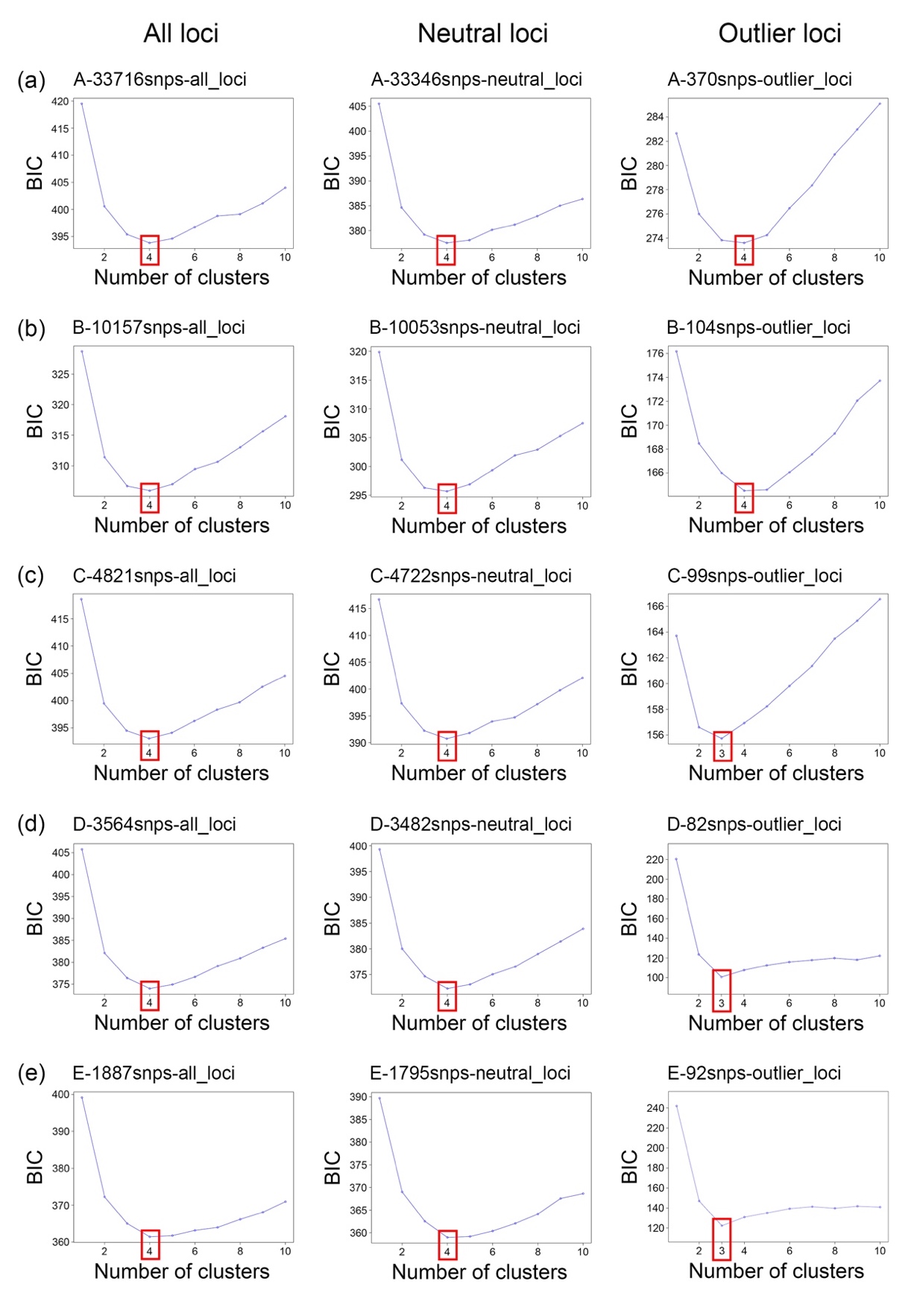


**Figure S3.** Plots of Bayesian Information Criterion (BIC) vs. number of clusters (*k*) of DAPC analyses for all, neutral, and outlier SNP loci from matrices A-E.


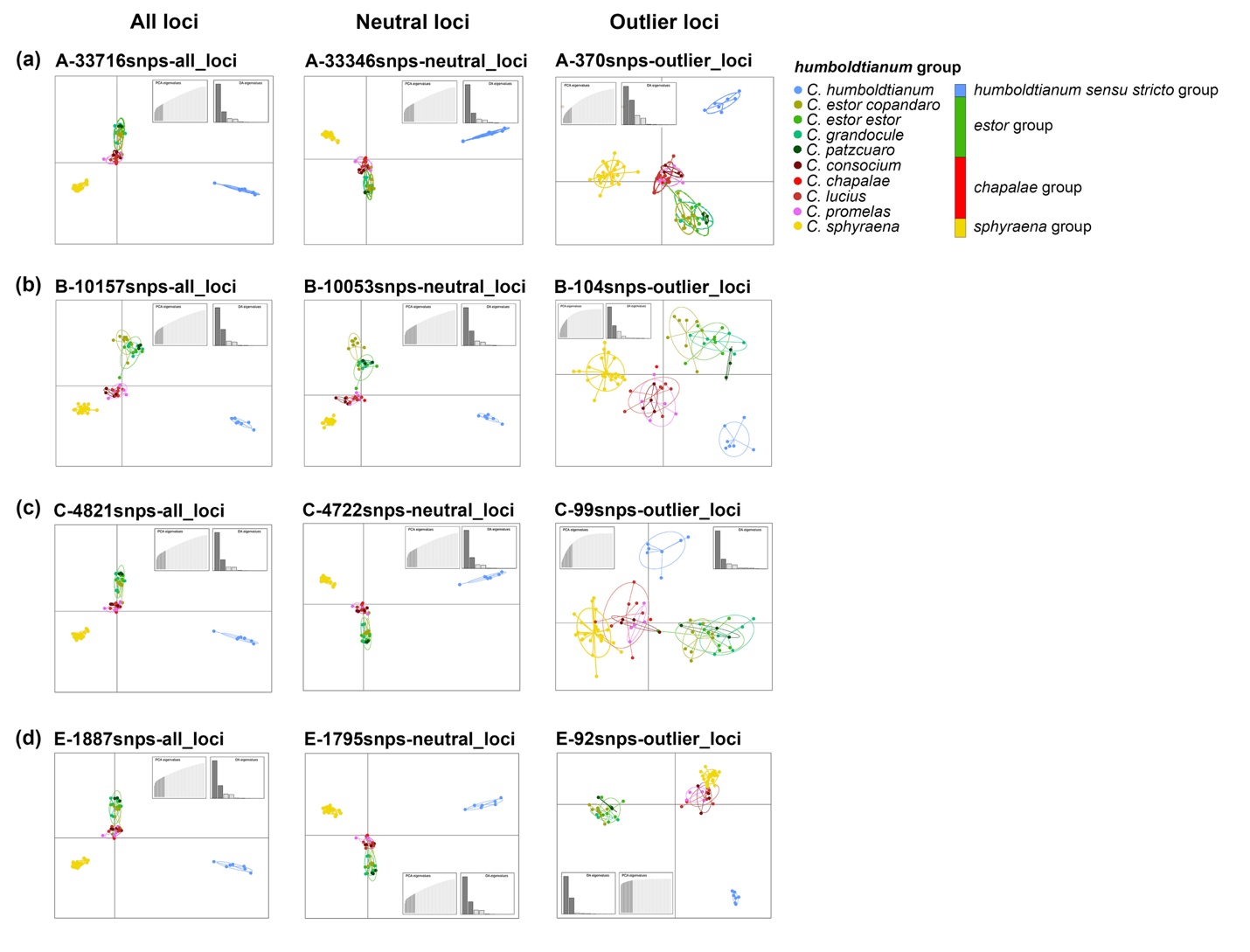


**Figure S4.** Discriminant analyses of principal components (DAPCs) estimated using *ca.* 1800–33700 SNPs resolve four well-differentiated clusters. DAPCs based on outlier SNPs recover three to four groups. These results are largely consistent across analyses based on matrices A, B, C, and E (a–d), and are also concordant with the PCA analyses. In neither case, the observed genomic clusters do correspond to the morphology-based species delimitation scenario. Morphospecies are color-coded according to the genomic clusters recovered: blue, *humboldtianum sensu stricto*, green, *estor* group; red, *chapalae* group; yellow, *C. sphyraena*.


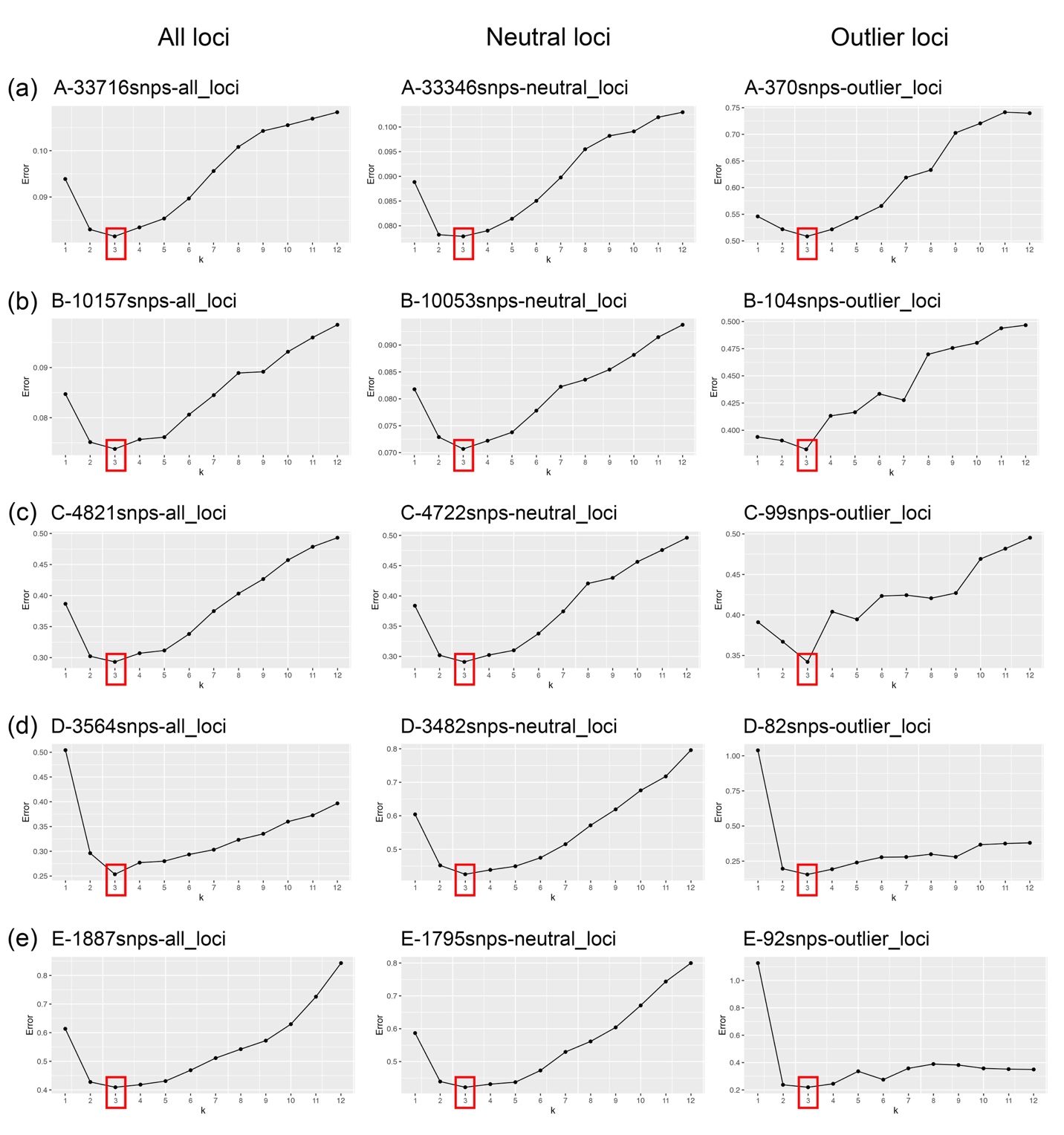


**Figure S5.** Plots of cross-validation error of each *k* (number of clusters) analyzed in Admixture analyses for all, neutral, and outlier SNP loci from matrices A- E. The cross-validation procedure was performed with the folds value = 5 (the default), a block relaxation algorithm as point estimation method, and the point estimation terminated with the objective function delta < 0.0001.


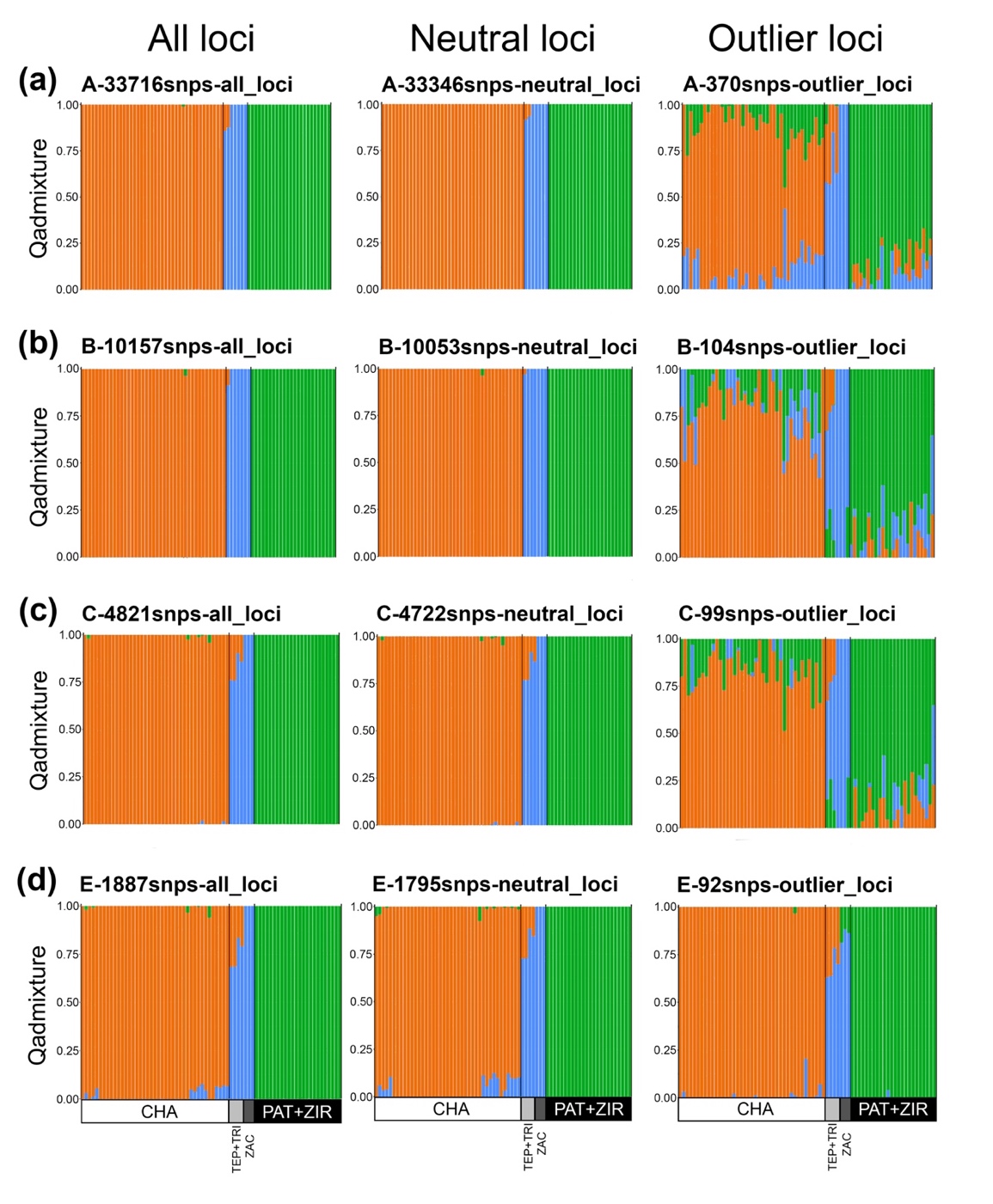


**Figure S6.** Admixture assignment analyses estimated using *ca.* 80–33700 neutral and outlier SNPs consistently identified three well-differentiated clusters (*k* = 3) using matrices A, B, C, and E (a–d). Outlier SNPs show an increased intermingling of individuals among clusters compared with analyses based on all or neutral loci. Each bar represents the probability of assignment to each cluster. Genomic clusters are color-coded as blue, *humboldtianum sensu stricto*, green, *estor* group; orange, *chapalae-sphyraena* group. CHA, Lake Chapala; TEP, Tepuxtepec Dam; TRI, Trinidad Fabela Dam; PAT, Lake Pátzcuaro; ZIR, Lake Zirahuén.


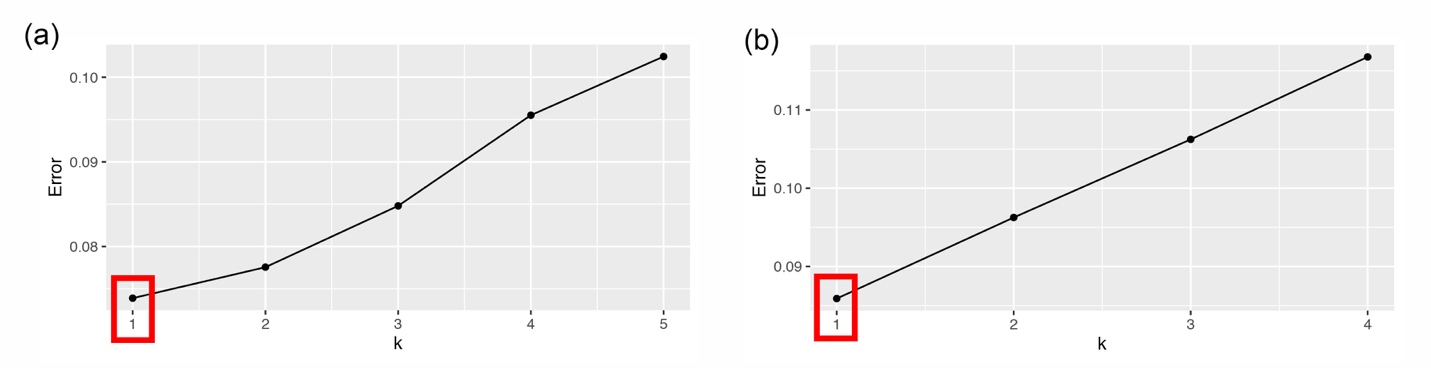


**Figure S7.** Admixture assignment analyses estimated using *ca.* 33700 SNP loci a) in Chapala Lake, and b) within Lakes Pátzcuaro-Zirahuén.


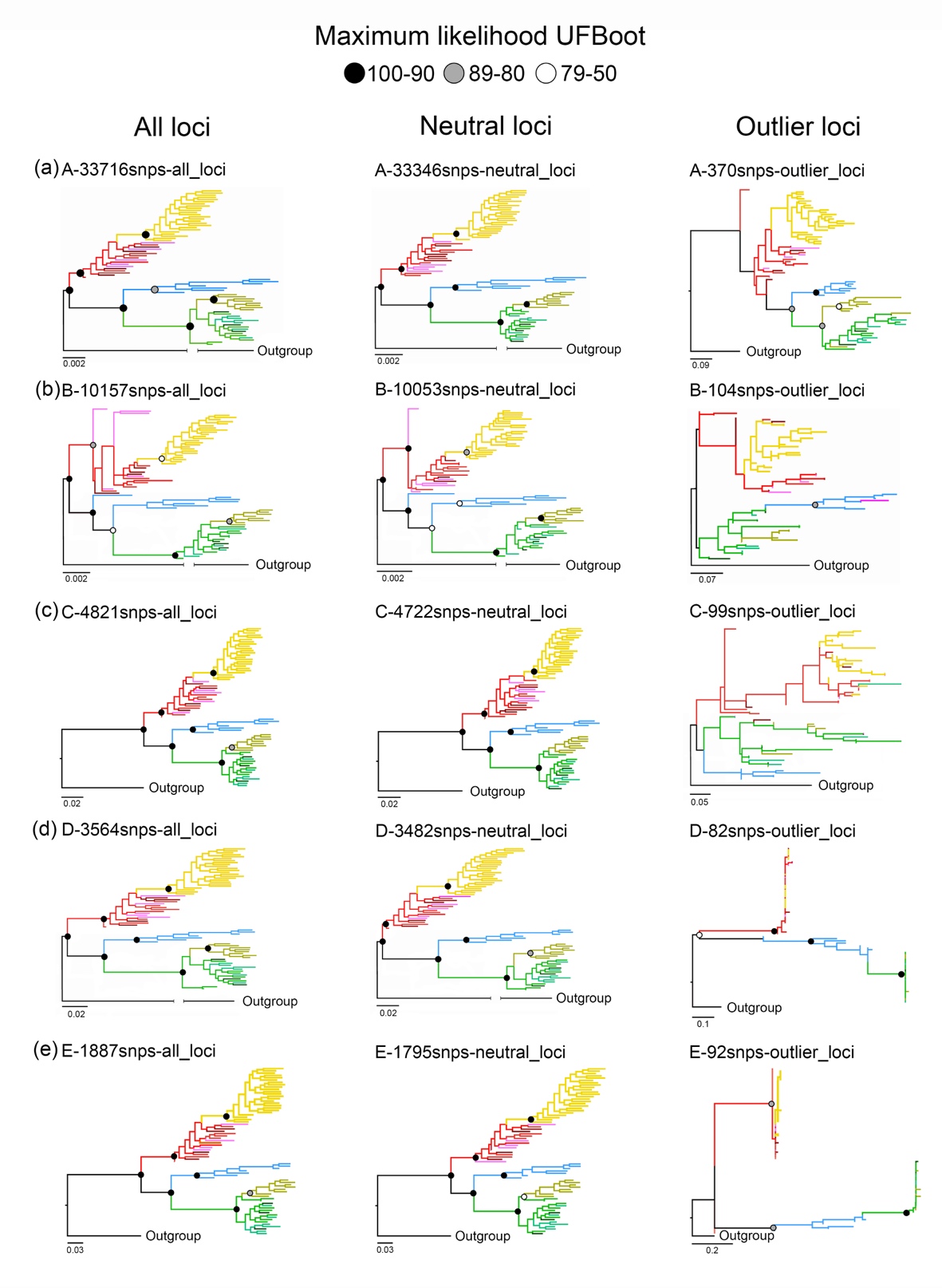


**Figure S8.** Phylogenetic trees of *ca.* 1800–33700 SNP loci of the *humboldtianum* group estimated under a maximum likelihood framework in IQ-TREE. Phylogenetic trees were estimated using all (~1900–33700), neutral-only (~1800–33300), and outlier (~80–350) SNPs. Individuals are color-coded by genetic clusters according to Fig. 2.


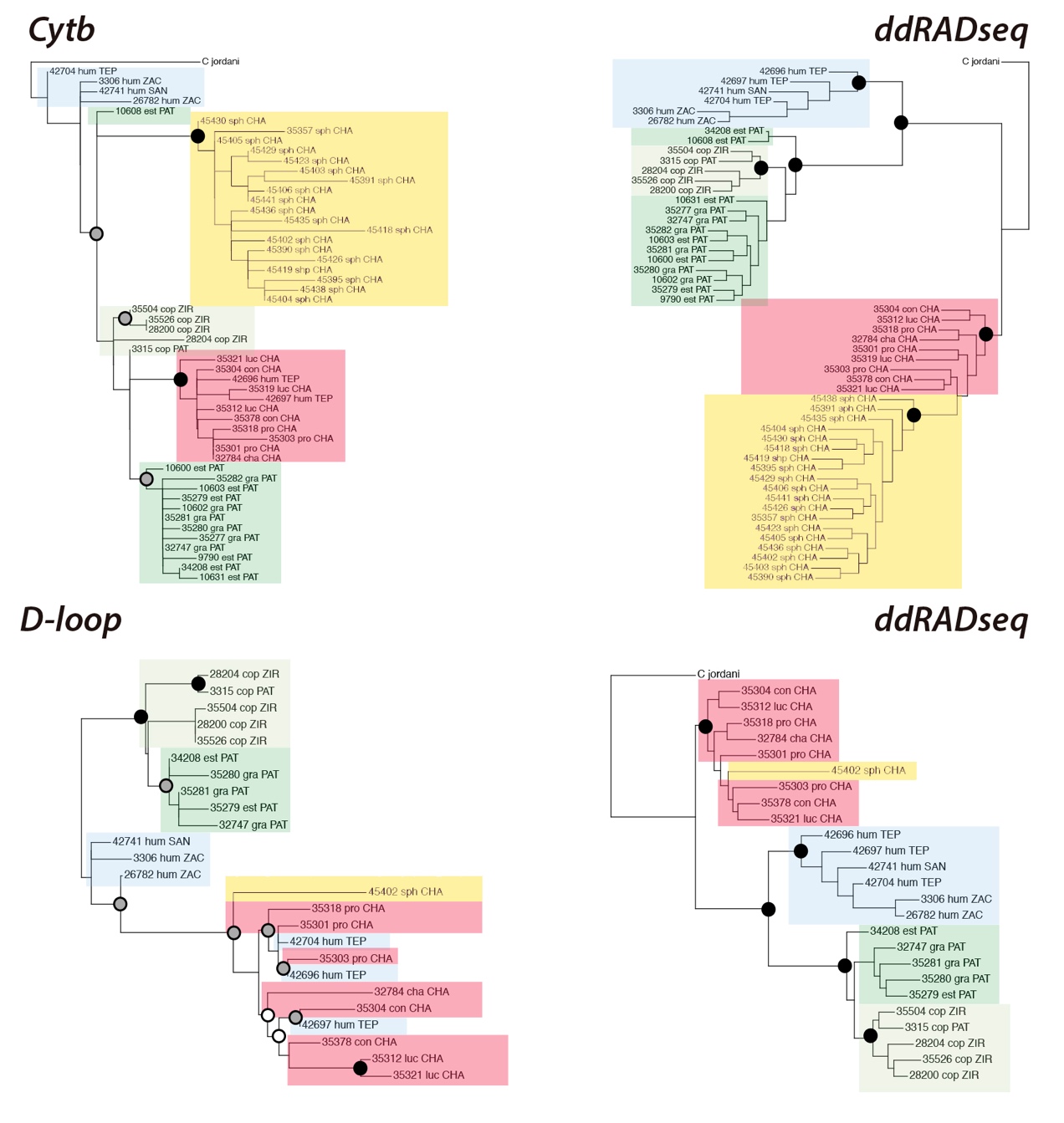


**Figure S9.** Mitochondrial trees (left) and ddRADseq phylogeny (right) of the *humboldtianum* group. The tips in the RADseq inferences were pruned to include the same individuals present in the mitochondrial trees.

**
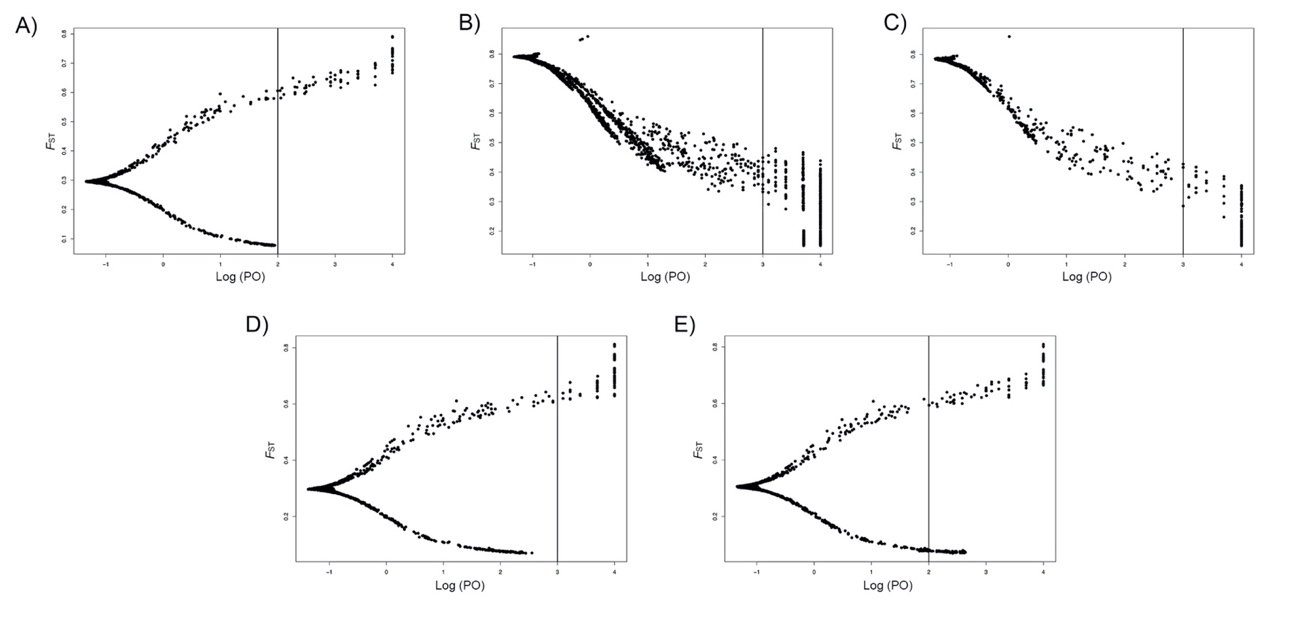
**

**Figure S10.** *F*_ST_ *versus* log10-transformed posterior odds (PO) values for the global outlier detection calculated in BayeScan. The analyses were estimated considering the nine morphospecies and (A) 33716 SNPs, where the vertical line represent the FDR threshold of *q*=0.037; (B) 10157 SNPs, *q*=0.040; (C) 4821 SNPs, *q*=0.025; (D) 3564 SNPs, *q*=0.034; and (E) 1887 SNPs, *q*=0.015.


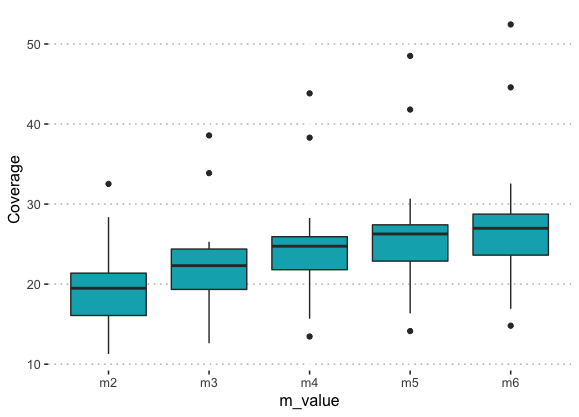


**Figure S11.** Mean coverage of the subset with 15 samples using different values of the minimum raw reads required to form a stack (*m*1–*m*6).


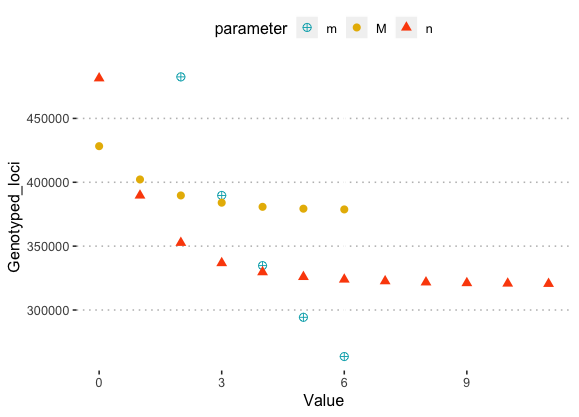


**Figure S12.** Putative loci at different combinations of *de novo* assembly parameters: *m*, minimum reads required to form a stack; *M*, allowed SNPs in a stack required to form a putative locus in an individual; *n*, allowed SNPs in a stack required to form a locus in the population. Each parameter was changed one at the time (*m* = 2–6, *M* = 0–6, and *n* = 0–11) while keeping the others at default values (*m*3*M*2*n*1).

**Figure S13.** Genotyped loci and variant sites using a constraint on the number of minimum individuals in a population required to have that locus (r = 40, 60, and 80) based on different cutoff, as follow: (A) minimum raw reads required to form a stack (*m* = 2–6), (B) maximum mismatches allowed between stacks of the same individual (*M* = 0–6), and (C) mismatches allowed between loci of different individuals (*n* = 0–11).

**
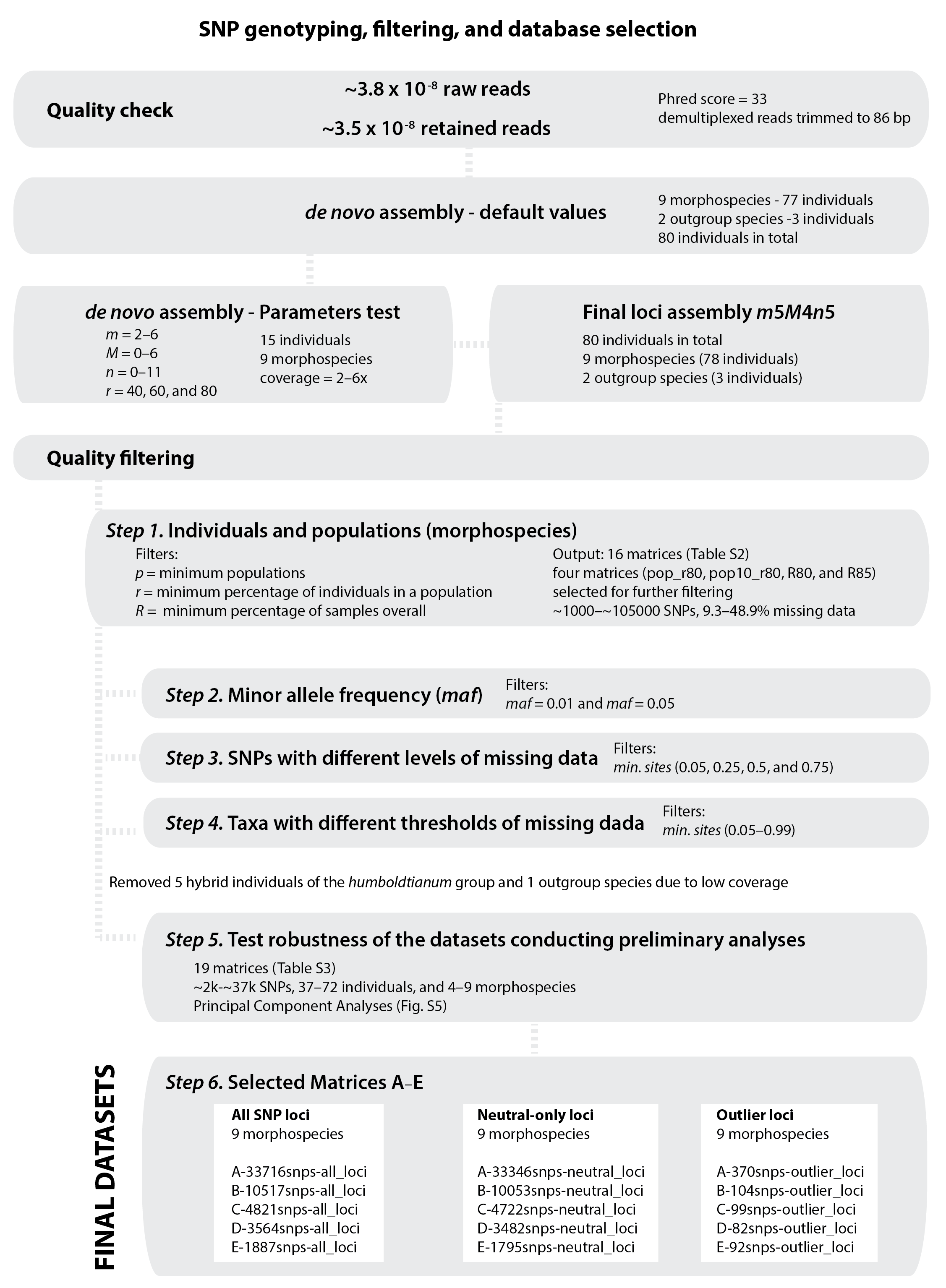
**

**Figure S14.** Flow chart of the quality control and SNP filtering steps applied to generate the final datasets.

**Supplementary References**

1. Barbour CD. The systematics and evolution of the genus Chirostoma Swainson (Pisces, Atherinidae). Tulane Stud Zool Bot. 1973;18:97–141. https://www.biodiversitylibrary.org/part/11912.

2. Peterson BK, Weber JN, Kay EH, Fisher HS, Hoekstra HE. Double digest RADseq: An inexpensive method for de novo SNP discovery and genotyping in model and non-model species. PLoS One. 2012;7.

3. Catchen JM, Hohenlohe PA, Bassham S, Amores A, Cresko WA. Stacks: an analysis tool set for population genomics. Mol Ecol. 2013;22:3124–40.

4. Catchen JM, Amores A, Hohenlohe P, Cresko W, Postlethwait JH. Stacks : Building and Genotyping Loci De Novo From Short-Read Sequences. Genes, Genomes. 2011;1 August:171–82.

5. Paris JR, Stevens JR, Catchen JM. Lost in parameter space: A road map for Stacks. Methods Ecol Evol. 2017;8:1360–73.

6. Mastretta-Yanes A, Arrigo N, Alvarez N, Jorgensen TH, Piñeros D, Emerson BC. Restriction site-associated DNA sequencing, genotyping error estimation and de novo assembly optimization for population genetic inference. Mol Ecol. 2015;:28–41.

7. Del Pedraza-Marrón CR, Silva R, Deeds J, Van Belleghem SM, Mastretta-Yanes A, Domínguez-Domínguez O, et al. Genomics overrules mitochondrial DNA, siding with morphology on a controversial case of species delimitation. Proc R Soc B Biol Sci. 2019;286.

8. Danecek P, Auton A, Abecasis G, Albers CA, Banks E, DePristo MA, et al. The variant call format and VCFtools. Bioinformatics. 2011;27:2156–8.

9. Linck E, Battey CJ. Minor allele frequency thresholds strongly affect population structure inference with genomic data sets. Mol Ecol Resour. 2019;19:639–47.

10. Bradbury PJ, Zhang Z, Kroon DE, Casstevens TM, Ramdoss Y, Buckler ES. TASSEL: Software for association mapping of complex traits in diverse samples. Bioinformatics. 2007;23:2633–5.

11. Betancourt-Resendes I, Perez-Rodríguez R, Barriga-Sosa IDLA, Piller KR, Domínguez-Domínguez O. Phylogeographic patterns and species delimitation in the endangered silverside “humboldtianum” clade (Pisces: Atherinopsidae) in central Mexico: understanding their evolutionary history. Org Divers Evol. 2020;20:313–30.

12. Kass RE, Raftery AE. Bayes Factors. J Am Stat Assoc. 1995;90:773–95.
